# Quantification of membrane fluidity in bacteria using TIR-FCS

**DOI:** 10.1016/j.bpj.2024.06.012

**Published:** 2024-06-13

**Authors:** Aurélien Barbotin, Cyrille Billaudeau, Erdinc Sezgin, Rut Carballido-López

**Affiliations:** 1 Université Paris-Saclay, INRAE, AgroParisTech, Micalis Institute, Jouy-en-Josas, France; 2 Science for Life Laboratory, Department of Women’s and Children’s Health, Karolinska Institutet, Solna, Sweden

## Abstract

Plasma membrane fluidity is an important phenotypic feature that regulates the diffusion, function, and folding of transmembrane and membrane-associated proteins. In bacterial cells, variations in membrane fluidity are known to affect respiration, transport, and antibiotic resistance. Membrane fluidity must therefore be tightly regulated to adapt to environmental variations and stresses such as temperature fluctuations or osmotic shocks. Quantitative investigation of bacterial membrane fluidity has been, however, limited due to the lack of available tools, primarily due to the small size and membrane curvature of bacteria that preclude most conventional analysis methods used in eukaryotes. Here, we develop an assay based on total internal reflection-fluorescence correlation spectroscopy (TIR-FCS) to directly measure membrane fluidity in live bacteria via the diffusivity of fluorescent membrane markers. With simulations validated by experiments, we could determine how the small size, high curvature, and geometry of bacteria affect diffusion measurements and correct subsequent measurements for unbiased diffusion coefficient estimation. We used this assay to quantify the fluidity of the cytoplasmic membranes of the Gram-positive bacteria *Bacillus subtilis* (rod-shaped) and *Staphylococcus aureus* (coccus) at high (37°C) and low (20°C) temperatures in a steady state and in response to a cold shock, caused by a shift from high to low temperature. The steady-state fluidity was lower at 20°C than at 37°C, yet differed between *B. subtilis* and *S. aureus* at 37°C. Upon cold shock, the membrane fluidity decreased further below the steady-state fluidity at 20°C and recovered within 30 min in both bacterial species. Our minimally invasive assay opens up exciting perspectives for the study of a wide range of phenomena affecting the bacterial membrane, from disruption by chemicals or antibiotics to viral infection or change in nutrient availability.

## Significance

Using fluorescence correlation spectroscopy (FCS) with total internal reflection fluorescence (TIRF) illumination, we measured the diffusion speed of fluorescent membrane markers as a readout for membrane fluidity of growing *B. subtilis* and *S. aureus* cells. Monitoring the effect of cold shock provided unique information about the dynamics of the plasma membrane of these two bacterial species. The unprecedented capability of TIR-FCS to quantify the fluidity of a single membrane in living bacteria opens the door to a whole set of new studies that will shed light on the mechanisms underlying bacterial membrane homeostasis and its interactions with the environment.

## Introduction

The plasma membrane, also called the cell or cytoplasmic membrane, is a component of every living cell. It is a fluid mixture of lipids and proteins that separates the intracellular and extracellular spaces. The fluidity (inverse of the viscosity) of the plasma membrane is the physical parameter that defines how fast a given element can diffuse within the membrane at a given temperature. Thus, membrane fluidity is of utmost interest for protein diffusion and biomolecular interactions (1,2). Membrane fluidity also affects protein folding (3,4,5). In bacteria, membrane fluidity is known to vary in response to chemical (6), biochemical (7,8), and osmotic (9,10) stresses. Membrane fluidity has been shown to be critical in both Gram-negative bacteria (for respiration in *Escherichia coli* (11) and multidrug transport in *Methylobacterium extorquens* (12)) and Gram-positive bacteria (e.g.,., resistance to antibiotics in *Staphylococcus aureus* (13), adaptation to extreme growth conditions in *Staphylococcus haemolyticus* (14), and response to antimicrobial peptides in *Bacillus subtilis* (15)). Another hint of the importance of membrane fluidity in bacterial cells lies in the widespread existence of control mechanisms that maintain it by modifying lipid and protein composition (16,17). In particular, the fatty acid composition of phospholipids—the main class of lipids of the plasma membrane—is modified in response to changes in temperature to modulate steric constraints and thereby lipid packing (18). Furthermore, proteins such as flotillins (19,20) and MreB (21) are also thought to play a role in membrane fluidity in *B. subtilis*. In the case of extremophiles, the generation of exopolymers (22), cryoprotectants, and antifreeze proteins (23) are used to protect the bacterial membrane against changes in temperature.

Existing methods to characterize membrane fluidity in live and synthetic membranes include electron spin resonance (24,25) and NMR spectroscopy (19), membrane fatty acid analysis (9,13,24), fluorescence assays using environment-sensitive probes such as diphenylhexatriene (DPH), or ratiometric probes such as Laurdan (6), and the measurement of the diffusion speed of a fluorescent tracer using either single-particle tracking (SPT) (26), fluorescence recovery after photobleaching (27), or fluorescence correlation spectroscopy (FCS) (28).

In microbiology, the most frequently used techniques are fatty acid analysis and DPH anisotropy or ratiometric imaging with environment-sensitive probes (typically Laurdan). Fatty acids analysis partially informs on the membrane composition but is an indirect readout of fluidity. It gives multidimensional results (relative proportions of different fatty acids with branching and (poly)unsaturation), which can be challenging to directly associate with a change in membrane fluidity, and can in the best case only provide qualitative comparisons of the resulting fluidity. Environment-sensitive fluorescent probes can give useful insights but can only measure relative differences in membrane fluidity. Many probes exist that are sensitive to different parameters of the membrane (29,30) and their behavior can be biased by unforeseen interactions (31).

FCS has occasionally been used in a few instances in bacterial membranes, to study protein diffusion (32,33,34), RNA concentration (35), assembly of protein complexes (36), or membrane dynamics in response to antibiotic treatment (28). These studies were all performed using confocal microscopy, the axial resolution of which (∼600 nm) is not well suited for measurements in bacteria. The axial resolution of confocal microscopes is comparable with the diameter of most studied bacterial cells (500 nm–1 *μ*m), and this results in problems such as such as having both top and bottom membranes in focus at once or excessive background from out-of-focus membranes. These limitations can be overcome either by using super-resolution microscopy (37) or more simply by using total internal reflection fluorescence (TIRF) microscopy. TIRF microscopy significantly improves the axial resolution of a microscope by illumination with an evanescent field that usually significantly decays within a range of 100 nm at the interface between the coverslip and the sample, which makes it ideally suited for the investigation of events at the cell surface.

Total internal reflection-fluorescence correlation spectroscopy (TIR-FCS) (38,39) was previously used to study molecular dynamics in eukaryotic cells (40,41) and to measure the fluidity of flat synthetic membranes (42,43). However, TIR-FCS was not previously applied to bacteria despite TIRF having become common for the study of molecular dynamics in the bacterial membrane (44). TIR-FCS offers several advantages over confocal FCS, besides the unrivalled axial selectivity of TIR illumination. First, camera-based TIR-FCS also offers massive parallelization of measurements: hundreds of FCS curves can be acquired simultaneously instead of a single one on a confocal microscope. Second, TIR-FCS can easily generate diffusion maps and therefore retrieve spatial information. Finally, TIR-FCS enables, by resampling intensity fluctuations in space after acquisition, the measurement of diffusion speeds at different spatial scales (spot-variation FCS (45)).

Here, we extend the scope of application of TIR-FCS to measure membrane fluidity in live bacterial cells with different morphologies, exemplified by the Gram-positive leading model organism *B. subtilis* and the pathogen *S. aureus*, which are respectively rod-shaped and spherical. To simplify data analysis and fully use the high-throughput capability of imaging FCS, we developed a new FCS quality metric to automatically discard artifactual curves. Using simulations validated by experiments in synthetic samples, we measured the bias induced by the small size and curvature of bacterial membranes on TIR-FCS measurements. We demonstrated the validity of our assay by studying the well-known response of the *B. subtilis* plasma membrane to a cold shock (46,47) and the much less-studied cold shock response of *S. aureus*. Diffusion measurements of the membrane markers Nile red and Di4-ANEPPS confirmed the previous knowledge about cold shock recovery in *B. subtilis* and provided unprecedented insights of bacterial membrane dynamics at different temperatures in both *B. subtilis* and *S. aureus*.

## Materials and methods

### FCS setup

TIR-FCS acquisitions were performed on a Zeiss Elyra PS1 microscope equipped with a 100×/1.46 NA Apochromat oil immersion objective. A typical FCS acquisition consisted of 50,000 frames, on a field of view of 128 × 10 pixels with a pixel size of 160 nm in the object plane and a frame acquisition time of 1.26 ms, the maximum achievable with our camera. Stable focus was ensured using Definite Focus. Detection was performed using an emCCD camera (Andor iXon 897), using maximum preamplification (5×) and electron-multiplying gains (300×) settings. Laser excitation at 561 nm was set, unless specified otherwise, to 5% of the maximum excitation power in *B. subtilis* and synthetic samples and 10% in *S. aureus.* 5% of the maximum excitation corresponded to a power of 460 *μ*W in epifluorescence mode measured in the focal plane of the objective. The excitation area was of approximately 80 × 80 *μ*m size, leading to an estimated power density of ∼70 nW/*μ*m^2^.

### Data processing and fitting

Pixels from each image stack were numerically binned 2 by 2, unless specified otherwise. The first 1500 frames of every acquisition were discarded as an occasional loss of focus could lead to artifactual intensity fluctuations in these frames. Intensity timetraces at each binned pixel were corrected for bleaching using a double exponential fit (48). Intensity traces at each binned pixel were correlated using a python implementation of the multipletau algorithm (49). FCS curves were fitted using the standard two-dimensional (2D) imaging FCS model (50) (except in *B. subtilis* cells where the fitting model is described in impact of membrane curvature, Eq. 5):

(1) $$\begin{array}{c} g_{xy}\left(\tau \right)=\frac{1}{N}\left(\frac{1}{\sqrt{\pi }\mu }\left(\exp\left(-\mu ²\right)-1\right)+\mathrm{erf}\left(\mu \right)\right)²\\ \mu =\frac{a}{2\sqrt{\sigma ²+D\tau }} \end{array}$$

where *N* is the average number of molecules in the observation area, *a* is the effective pixel size (320 nm with 2 × 2 pixel binning), *D* the diffusion coefficient, *τ* the lag time, and σ is the standard deviation of the microscope’s point spread function (PSF) approximated as a 2D Gaussian function:

(2) $$PSF\left(x,y\right)=\exp\left(-\frac{\left(x²+y²\right)}{2\sigma ²}\right)$$

Calibration of the *PSF* was done as described in (51). We measured our *PSF* σ = 0.19 *μ*m, corresponding to a fullwidth at half-maximum (FWHM) of 450 nm, larger than expected using a 1.46 NA oil immersion objective. This enlargement was likely caused by our use of a low-magnification tube lens that degraded resolution, as we measured a *PSF* size σ = 0.16 *μ*m when using a higher magnification tube lens. For all acquisitions except in supported lipid bilayers (SLBs), we used an intensity threshold set to 80% of the maximum intensity in the field of view (see supporting material for the determination of intensity threshold).

### Liposomes preparation

1,2-di-(9Z-Octadecenoyl)-*sn*-glycero-3-phosphocholine (DOPC) and 1-palmitoyl-2-oleoyl-*sn*-glycero-3-phosphocholine (POPC) stored in chloroform were purchased from Merck (Darmstadt, Germany) and stored under argon. Fifty microliters of 10 mg/mL stock were added to a glass tube then dried using argon under rotation. Lipids were resuspended in 1.6 mL phosphate-buffered saline (PBS), then tip-sonicated for 10 min in 30 s on/off cycles on ice. Liposomes were labeled before experiments with 1% of 1,2-dioleoyl-*sn*-glycero-3-phosphoethanolamine-*N*-(lissamine rhodamine B sulfonyl) (PE-Rhod) (Merck) at a concentration of 10 *μ*g/mL.

### SLB preparation

SLBs were prepared by liposome deposition. We pipetted 20 *μ*L of liposome solution to a home-made microfluidic chamber made of a sandwich of a slide and a plasma-cleaned coverslip held together by two strips of molten parafilm. Excess liposomes were abundantly washed using 200 *μ*L PBS. The chamber was then sealed using parafilm to prevent evaporation.

### Beads-supported lipid bilayer preparation

Beads-supported lipid bilayers (BSLBs) were prepared as described elsewhere (52). Ten microliters of 5 *μ*m uncoated silica beads (BioValley, Nanterre, France) were washed twice in 1 mL PBS, then mixed with 50 *μ*L liposomes. The mix was shaken for 20 min to form BSLBs, then washed twice in 1 mL PBS. PBS (200 *μ*L) was left after final wash, and 100 *μ*L of BSLBs was then pipetted to a glass-bottom Ibidi chamber for imaging.

### Cells preparation and staining

#### B. subtilis

The wild-type laboratory strain 168 *trpC2* of *B. subtilis* was grown and imaged in rich lysogeny broth medium (LB). For measurements at 37°C, 3 *μ*L of cells from an overnight culture grown at 30°C were diluted in 2 mL LB and grown at 37°C under agitation for 2.5 h until they reached exponential phase (OD_600_ ∼ 0.3) and then labeled with 0.2% (v/v) of either 50 *μ*g/mL Nile red or 40 *μ*g/mL Di4-ANEPPS (Thermo Fisher, Waltham, MA, USA) dissolved in DMSO (see Fig. S1 for a discussion on labeling concentration). Labeled cultures were left under agitation for an additional 15–20 min, then 3 *μ*L was transferred to an agarose pad (1.2% in LB) for immobilization and covered with a plasma-cleaned coverslip.

For steady-state measurements at 20°C, cells from an overnight culture at 30°C were first diluted (3 *μ*L of overnight culture diluted in 2 mL fresh LB) and grown for about 2 h at 37°C until reaching exponential phase (OD_600_ ∼ 0.2), transferred at 20°C under agitation for 4–5 h for at least one generation, then labeled as described above.

#### S. aureus

The attenuated wild-type laboratory strain of *S. aureus* RN-4220 (53) was prepared similarly to *B. subtilis*, except for the following points. At 37°C, cells were grown until reaching exponential phase (OD_600_ ∼ 0.3–0.4). Cells were labeled with 0.2% of a stock of 20 *μ*g/mL of Nile red, lower than in *B. subtilis* as we observed phototoxicity when performing FCS experiments in *S. aureus* with a stock concentration of 50 *μ*g/mL. For steady-state measurements at 20°C, cells were grown at 37°C to an OD_600_ of ∼0.2, transferred at 20°C for 3 h for at least one generation.

#### Delay before acquisition

When *B. subtilis* cells were transferred from the liquid culture to the agarose-coated slide at the same temperature, we observed an initial decrease in membrane fluidity followed by membrane fluidity recovery within about 25 min (Fig. S2). This adaptation to the transfer on agarose pad could be due to osmotic shock (54), oxidative stress, or another cause that remains unknown. We therefore leave cells (both *B. subtilis* and *S. aureus*) to settle on the agarose-coated slide for 25 min before starting to image. In the case of cold shock, cells were first immobilized on an agarose pad and covered with the coverslip at 37°C for 25 min then transferred at 20°C in the microscope.

## Results

### Filtering curves based on goodness of fit

FCS measurements can be subject to artifacts that distort FCS curves, for instance, when bright clusters of fluorescent molecules enter the observation area. These curves need to be discarded from the analysis to avoid biasing the estimation of the diffusion coefficient. In point FCS, this is often done via manual inspection facilitated by dedicated software (55). This approach is, however, impractical in imaging FCS due to the high parallelization of FCS curve acquisition resulting in the generation of a high number of FCS curves (typically we acquire 350–500 FCS curves per hour in bacteria, considering only one pixel binning value). The issue of sample-induced artifacts in FCS is well acknowledged and solutions to this issue were developed previously. It was notably proposed to compare each curve within a data set to the averaged curve and to exclude outliers (56). This solution is, however, computationally intensive and might fail if signal levels vary within a data set, for instance, due of cell-to-cell heterogeneity. Another approach consists in rejecting curves with irregular residuals. One method for doing this consists in calculating the χ^2^ goodness of fit (32), but it does not handle noisy curves well (56). It also requires knowledge of the standard deviation of the FCS curve, which is not always available. The Fourier transform was previously used to detect unevenly distributed residuals, relying, however, on fine-tuning three empirical parameters and making assumptions on the transit times observed (35). As an alternative to these methods, we introduce here a simple error metric based on fitting residuals that quantifies fitting bias. Considering the mean square error (*MSE*):

(3) $$\begin{array}{c} MSE=\frac{1}{n}\sum_{i}^{n}{\left(r_{i}\right)}^{2}\\ r_{i}=\left(y_{i}-{ŷ}_{i}\right)/{ŷ}_{0} \end{array}$$

where *y*_*i*_ is the empirical FCS curve at lag time *I*, *ŷ*_*i*_ is the corresponding fit value and *r*_*i*_ the residual. A high *MSE* value is indicative of either a high fitting bias in a poorly fitted curve, which needs to be discarded, or of low signal value (57) caused by strong oscillations of the FCS curve around its fit. Expecting strong variations in signal levels within and across acquisitions, due to cell-to-cell variations and inhomogeneous illumination of curved cells in the TIRF field, we designed a metric that measures fit quality with a lower dependency to signal level. This metric, named nonlinear mean-square error (*MSE*_*nl*_), is a weighted sum of residuals, where the weight of each residual is equal to the number of adjacent residuals of same sign *n*_*adj*,*i*_:

(4) $${MSE}_{nl}=\frac{1}{n}\sum_{i}^{n}n_{adj,i}{\left(r_{i}\right)}^{2}$$

**Figure 1:**
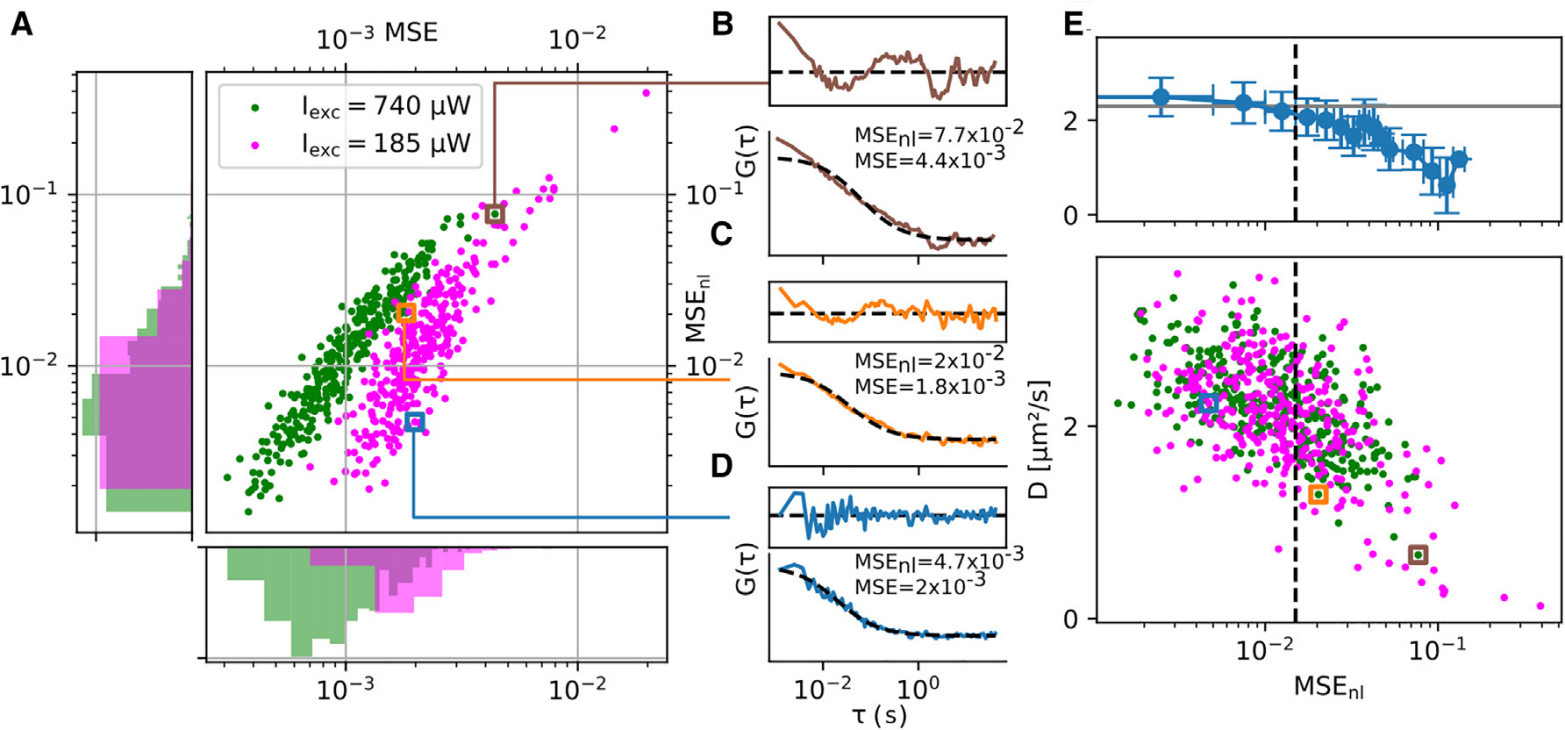
Estimation of the fit quality of FCS curves acquired on a DOPC SLB labeled with PE-Rhod using *MSE* and *MSE*_*nl*_. (*A*) Scatterplot (*center*) of *MSE* and *MSE*_*nl*_ metrics for each curve acquired in TIR-FCS at low (*magenta*) and high (*green*) excitation powers as indicated in the legend, and histograms of the corresponding *MSE* (*bottom*) and *MSE*_*nl*_ (*left*) distributions. Colored squares refer to FCS curves shown in (*B*)*–*(*D*). (*B–D*) Normalized FCS curves (*color*) and fits (*dotted black lines*) with (*top*) fitting residuals, all plotted with the same scale. (*C* and *D*) FCS curves with similar *MSE* and different *MSE*_*nl*_. (*B*) FCS curve with both higher *MSE* and *MSE*_*nl*_ than curves in (*C*) and (*D*). (*E*) Bottom: scatterplot of measured diffusion coefficient with *MSE*_*nl*_ and empirical threshold on fitting quality (*dotted vertical line*) to discard artifactual FCS curves. Squares represent FCS curves shown in (*B*)*–*(*D*). Top: mean ± SD of diffusion coefficient within the *MSE*_*nl*_ range for both high and low excitation intensities, represented by lateral error bars (*blue*) and (*gray*) value reported for the diffusion coefficient of PE-Rhod in a DOPC SLB in (58). To see this figure in color, go online.

Concretely, if residuals *r*_*i*_ between *i–2* and *i+2* are all positive, the fitting bias is strong and the weight *n*_*adj*,*i*_ = 4 is high. On the other hand, if the residual *r*_*i*_ is positive but the residuals *i–1* and *i+1* are negative, there is no fitting bias (residuals are oscillating around the mean) and *n*_*adj*,*i*_ = 0. To evaluate the capability of *MSE*_*nl*_ to evaluate fitting bias, we performed two different TIR-FCS acquisitions on a flat sample of DOPC SLBs, at either high (740 *μ*W) or low (185 *μ*W) excitation power. The resulting data set had heterogeneous signal levels representing the expected heterogeneity in biological samples. However, since these two acquisitions were performed on the same SLB, we expected to find a comparable number of artifactual curves with the two excitation intensities. By comparing *MSE* and *MSE*_*nl*_ for every FCS curve in the data set (Fig. 1 *A*), we could observe that excitation intensity was a good predictor of *MSE* but not of *MSE*_*nl*_. Curves acquired at higher excitation intensity had on average a lower *MSE* but similar *MSE*_*nl*_, showing a lower dependence of *MSE*_*nl*_ with signal levels. We observed a strong positive correlation between the two metrics for curves acquired at a same excitation intensity (Fig. 1 *A*). This is due to poorly fitted curves having higher residuals than well-fitted curves at similar signal levels (Fig. 1, *B* and *D*). However, comparing two FCS curves with similar *MSE* but different *MSE*_*nl*_ suggested that *MSE*_*nl*_ measures fitting bias irrespective of signal levels (Fig. 1, *C* and *D*), unlike *MSE* which is heavily affected by signal levels. Considering that every FCS curve contains imperfections, we then sought to determine the maximum acceptable amount of fitting bias measured by *MSE*_*nl*_. For this, we plotted the measured diffusion coefficient against *MSE*_*nl*_ (Fig. 1 *E*, *bottom*). We observed that high fitting biases were correlated to slower diffusion coefficients caused by artifacts in FCS curves, which could originate from temporary loss of focus or fluorescent clusters moving through the measurement area. We confirmed this by plotting the average diffusion coefficient for sets of FCS curves with *MSE*_*nl*_ thresholds (Fig. 1 *E*, *top*) and found that the average diffusion coefficient obtained with FCS curves having a low *MSE*_*nl*_ matched the diffusion coefficient previously measured on PE-Rhod in a POPC SLB (58). Using both inspection of individual FCS curves and the scatterplot shown in Fig. 1 *E*, we set the *MSE*_*nl*_ threshold to the value of 0.015. We kept this threshold throughout this study, in both synthetic and biological samples.

### Impact of membrane curvature

**Figure 2:**
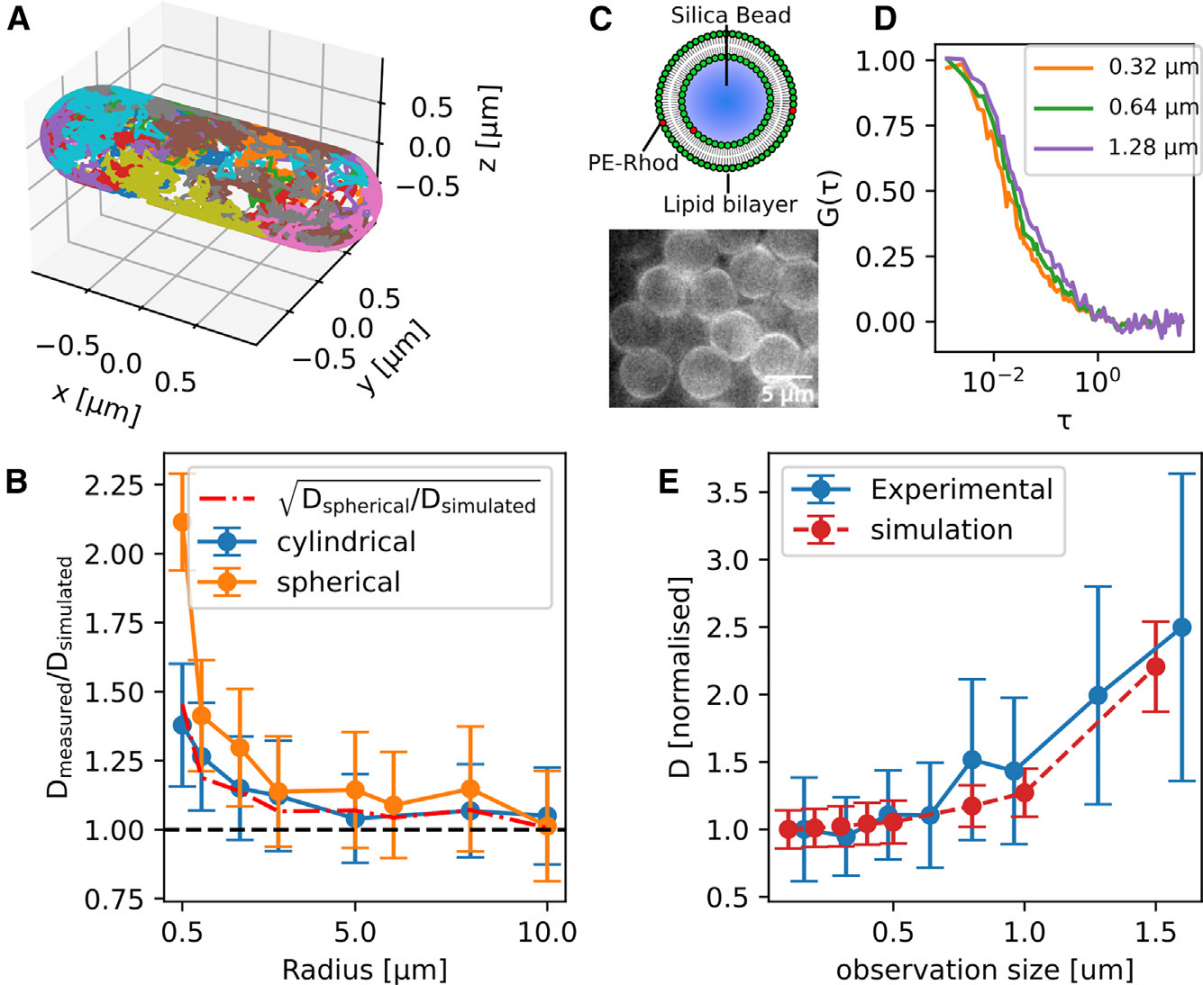
Impact of membrane curvature on TIR-FCS measurements. (*A*) 3D visualization of random trajectories generated on a rod. (*B*) Diffusion coefficients (mean ± SD) measured D_measured_ from simulation of FCS measurements, normalized with simulated value D_simulated,_ in cylindrical (*blue*) and spherical (*orange*) coordinates. Dash-dotted red line, square root of normalized diffusion coefficient in a sphere; dotted black line, simulated diffusion coefficient (ground truth). (*C*) Cartoon of a BSLB (*top*) and epifluorescence images of BSLBs in an Ibidi chamber (*bottom*). (*D*) FCS curves for different observation sizes (obtained by pixel binning) as indicated in the legend, averaged within a 1.28 × 1.28 *μ*m observation area. (*E*) Diffusion coefficients (mean ± SD) measured at different observation sizes, normalized with the value measured for smallest observation size from experimental measurements (*plain blue line*) in 5 *μ*m diameter BSLBs or from simulations in a sphere of same size (*dotted red line*). To see this figure in color, go online.

Three assumptions made when fitting FCS curves with the model in Eq. 1 were not verified when doing TIR-FCS in bacteria. First of all, Eq. 1 assumes that the diffusion within the observation area is occurring on a 2D flat surface. TIR-FCS measures an average transit time in the observation area and then calculates a diffusion coefficient as a ratio between the size of the observation area and the transit time. In Eq. 1, it is assumed that the size of the observation area is identical to the size of the area in which molecules diffused (the diffusion area). This is true when imaging a flat surface but is not when imaging a curved surface. In the latter case, the diffusion area is larger than the observation area. Second, it is assumed that intensity fluctuations are only caused by molecules moving across the observation area and Poisson noise. However, when doing TIR-FCS in a curved membrane, molecules moving laterally also change their axial position, which, under TIRF excitation, determines excitation intensity and therefore induces intensity fluctuations. The third assumption is that the system is open, which means that there is an infinite pool of fluorescent molecules diffusing in an infinite-sized reservoir. This latest assumption is never actually verified but it is a good approximation when the mean-square displacement of fluorescent emitters during the time of acquisition is much smaller than the reservoir size. This is not the case for membrane markers in bacteria: the average distance traveled by molecules diffusing at a reasonable 1 *μ*m^2^/s speed over the course of 1 min (our usual acquisition time) is 15 *μ*m, larger than the characteristic dimensions of a *B. subtilis* cell (typically ∼5 *μ*m long and ∼1 *μ*m wide for exponentially growing *B. subtilis* cells in LB medium at 37°C, see Fig. S3). To evaluate potential biases in diffusion coefficient measurements caused by these effects, we simulated diffusion on curved surfaces of finite areas: either on the simplest case of a sphere (that represents *cocci*, e.g., *S. aureus*) or on a cylindrical vessel (like the rod-shaped *B. subtilis*). Diffusion on these 3D surfaces were simulated as a Wiener process. We first generated a uniform distribution of initial positions. Position vectors were updated for each step by adding the cross-product of the position vector with a random 3D vector of Brownian motion, then normalized (see supporting material for a complete description of the simulation). From the position of individual emitters determined as trajectories (Fig. 2 *A*), we could simulate TIR-FCS experiments having the physical parameters of our setup: framerate (1 ms/frame), PSF size (FWHM of 450 nm), TIRF penetration depth (100 nm). Molecular brightness was set to 20 kHz and simulated diffusion coefficient was set to 1 *μ*m^2^/s. The number of molecules was set to reach a density of 0.4 molecules/*μ*m^2^, with a minimum of 10 molecules per simulation.

Within this simulation framework, we simulated a series of TIR-FCS experiments either on rods (of constant length set to 3 *μ*m) or on spheres of various radii (0.5–10 *μ*m) and measured the apparent diffusion coefficient (Fig. 2 *B*). We found that, in both geometries, the measured diffusion coefficient converged toward the real value for high radius values, which was expected given that a curved membrane of high radius of curvature can be approximated as flat. The asymptotic value was nonetheless a few percent higher than the target value, which could be due to the limited number of frames or limited surface area simulated. However, we found a significant bias in diffusion coefficient measurement for small radii (below 2 *μ*m). The measurement bias in a rod was well approximated by the square-root of the measurement bias in a sphere (Fig. 2 *B*, *dash-dotted line*). This can be explained by thinking of curvature as modifying the detection PSF: the detection PSF is modified alongside two dimensions in the case of a sphere and only one dimension in the case of a rod. FCS curves acquired in a rod shape can therefore be fitted with an updated model based on Eq. 1, which is the product of two 1D fitting models with a fitting bias *f* accounting for curvature alongside one dimension:

(5) $$\begin{array}{c} g_{xy},rod\left(\tau \right)=\frac{1}{a²}\left(\frac{1}{\sqrt{\pi }\mu_{1}}\left(\exp\left(-\mu_{1}²\right)-1\right)+\mathrm{erf}\left(\mu_{1}\right)\right)\left(\frac{1}{\sqrt{\pi }\mu_{2}}\left(\exp\left(-\mu_{2}²\right)-1\right)+\mathrm{erf}\left(\mu_{2}\right)\right)\\ \mu_{1}=\frac{a}{2\sqrt{\sigma ²+D\tau }};\mu_{2}=\frac{a}{2\sqrt{\sigma ²+Df\tau }} \end{array}$$

We set the fitting bias *f* to the value of 2.1 for *B. subtilis*, corresponding to the bias induced by a curvature of 500 nm radius on a sphere (Fig. 2 *B*). We investigated using simulations whether rod length influenced the measurement of diffusion coefficient (Fig. S10), and found that for a radius of 500 nm only rod lengths below 2.5 *μ*m affected the diffusion measurements, which is below the lengths of *B. subtilis* cells we measured in this study (Fig. S3). To validate our simulations experimentally, we used POPC BSLBs of 5 *μ*m diameter, labeled with the fluorescent lipid PE-Rhod, as a system of 2D diffusion of a controlled diameter (Fig. 2 *C*). We performed a series of TIR-FCS experiments in these BSLBs and measured the diffusion coefficient with different observation sizes using different pixel binning values (Fig. 2 *D*). The apparent curvature increased as the pixel binning increased and this experiment was therefore a good proxy to measure the effect of different curvatures on measured diffusion coefficient. As expected, we observed an increase in diffusion coefficient with increased observation size, corresponding to an increased effect of curvature on the effective PSF. We observed a similar change in diffusion coefficient with observation size on simulated spheres of identical diameter (Fig. 2 *E*). This analysis assumed free diffusion occurring in BSLBs, which was previously shown to not be strictly true (52). Nanoscale hindrances were previously detected in BSLBs using super-resolution spectroscopy (52), but these were unlikely to affect diffusion measurements at the larger observation scales used here. Indeed, we observed that the measured diffusion coefficient was constant for pixel sizes between 160 and 480 nm (Fig. 2 *E*), consistent with a free diffusion approximation.

### Temperature-induced changes in membrane fluidity in *B. subtilis*

The fluidity of biological membranes heavily depends on the ambient temperature. Reducing temperature increases lipid order, thereby decreasing membrane fluidity, up to the point of phase transition from a fluid membrane to a gel-like structure (59). Poikilothermic (“cold-blooded”) organisms like bacteria that are naturally exposed to ample changes in temperature adapt their membrane composition to maintain fluidity to survive such changes. Bacterial membrane adaptation to low temperature has been widely studied. In the Gram-positive model organism *B. subtilis*, it primarily involves increasing the ratio of unsaturated (containing double bonds) and branched fatty acids (47,60) that have a higher melting temperature than their saturated and straight-chain counterparts, resulting in more fluid membranes. The plasma membrane of exponentially growing *B. subtilis* cells in rich medium contains only low amounts of unsaturated fatty acids (47,61). Upon cold shock, the two-component system DesK/DesR is activated by an increase of membrane thickness (62), which in turn triggers the expression of the *des* gene (6), coding for the fatty acid desaturase Des that desaturates fatty acids (60) immediately (<30 min) (46). Longer-term membrane readaptation involves branching instead of saturation and a decrease in average fatty acid chain length (47). The resulting changes in membrane fluidity can, however, not directly be calculated from the fatty acid chain composition alone, as other changes come into play and the membrane fluidification by fatty acid saturation and branching at low temperature is compensated to an unknown extent by a loss of fluidity caused by a temperature decrease.

**Figure 3:**
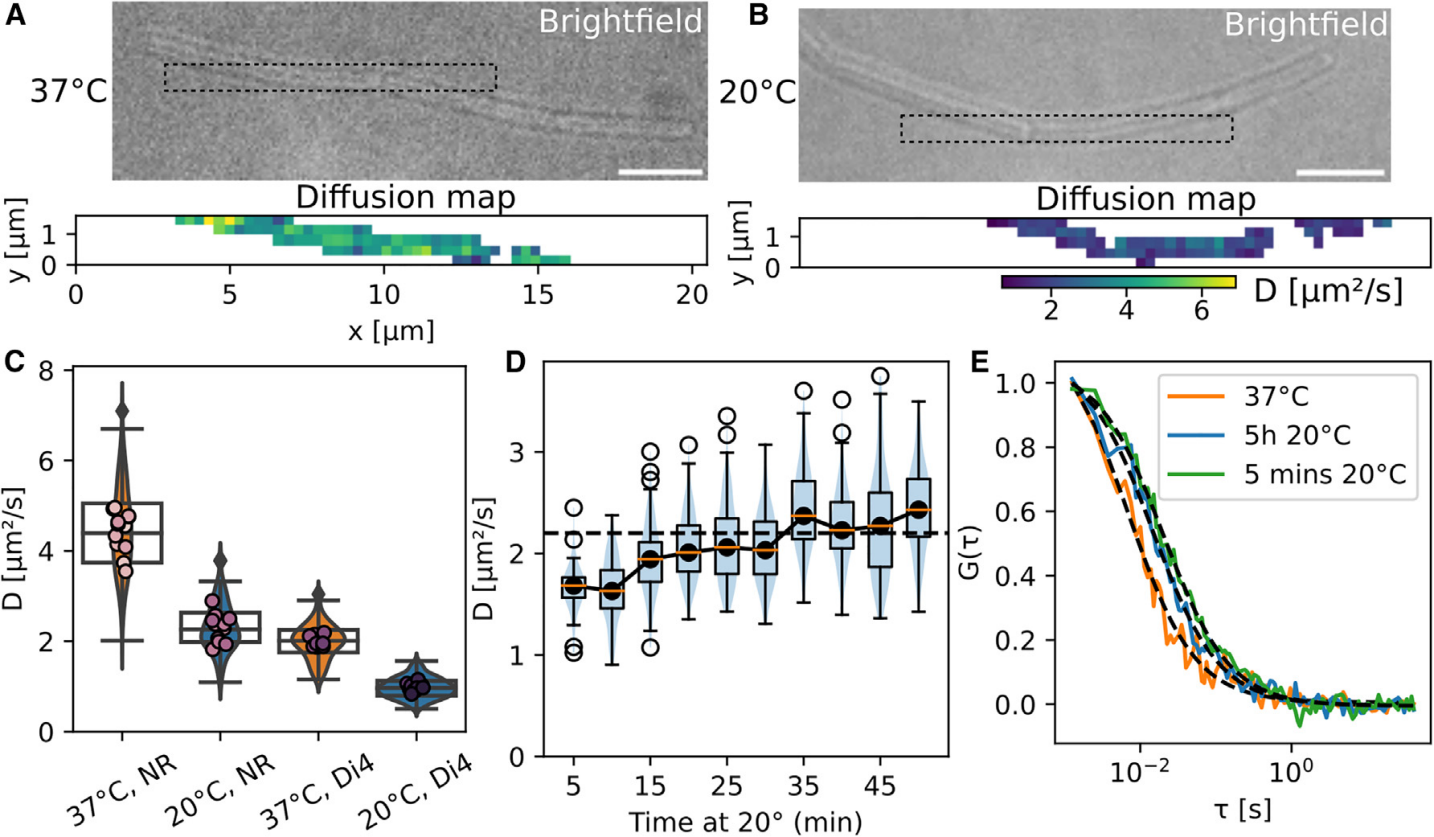
Adaptation of *B. subtilis* membrane to cold temperature, measured with TIR-FCS. (*A* and *B*) representative brightfield (*top*) with highlighted areas (*dotted squares*) where TIR-FCS diffusion maps (*bottom*) of Nile red in exponentially growing *B. subtilis* were acquired, at 37°C (*A*) or after 5 h at 20°C (*B*). Scale bars, 5 *μ*m. (*C*) Diffusion coefficient of Nile red (*left*) and Di4 (*right*) in *B. subtilis* cells grown and imaged at 37°C (*orange*) or 5 h after transfer at 20°C (*blue*). Dots: average of individual TIR-FCS acquisitions of one or more cells, two biological replicates, *n* > 280 FCS curves per condition. (*D*) Recovery of diffusion speed of Nile red upon transfer from 37 to 20°C, pooling measurements by tranches of 5 min, *n* = 2 biological replicates. Dotted black line: median diffusion coefficient of Nile red at 20°C from (*C*). (*E*) Normalized FCS curves obtained at 37°C (*orange*) and 5 min (*green*) and 5 h (*blue*) after transfer at 20°C. To see this figure in color, go online.

We thus aimed to measure membrane fluidity of *B. subtilis* cells growing at either 37 or 20°C using our TIR-FCS assay. We used the membrane dye Nile red, which is widely used in *B. subtilis* and has the advantage of being bright and photostable. Cells growing in liquid were stained, immobilized on an agarose-coated slide and allowed to stabilize for 25 min before imaging (see Fig. S2 and supporting material for details). Individual diffusion maps alone clearly showed that the diffusion speed of Nile red was slower at 20 than at 37°C (Fig. 3, *A* and *B*). This observation was verified through multiple acquisitions, showing an about twofold reduction in the diffusion coefficient of Nile red at 20°C relative to 37°C (Fig. 3 *C*). We verified that cells were healthy and exponentially growing by monitoring their growth before imaging (Fig. S4). The rare cells that were not growing in the microscopy field were excluded from the analysis. We also estimated the impact of phototoxicity by monitoring the growth at the single-cell level after imaging and found that cells were growing after imaging, although at a slightly slower rate (Fig. S4). To verify whether the diffusion speed of Nile red was controlled by membrane fluidity and not by unforeseen interactions, we performed the same experiment with the membrane dye Di4-ANEPPS (Di4). Diffusion speed of Di4 was slower than Nile red in similar experimental conditions (Fig. 3 *C*). This could be caused by its larger size (M = 318 g/mol for Nile red and 480 g/mol for Di4), different location in the membrane, or another unknown factor. The reduction of diffusion speed measured between 37 and 20°C was identical for both dyes (Fig. 3 *C*; Table 1), suggesting that it was indeed a change in fluidity that led to the observed reductions in diffusion coefficient.

**Table 1:** Diffusion coefficients of Nile red and Di4-ANEPPS measured in *B. subtilis* cells growing at 37 and 20°C, and ratios of diffusion coefficients at 37 and 20°C

Dye	D_37°C_ (mean ± SD)	D_20°C_ (mean ± SD)	D_37°C_/D_20°C_
Nile red	4.4 ± 0.3	2.2 ± 0.2	2 ± 0.3
Di4-ANEPPS	1.9 ± 0.1	0.9 ± 0.07	2.1 ± 0.3

Presented are the means and standard deviations of the average diffusion coefficients for each acquisition (*dots* in Fig. 3*C*).

Having established a baseline for the diffusivity of Nile red in *B. subtilis* at different temperatures, we then sought to monitor the remodeling of the membrane in response to a cold shock. Cells growing exponentially at 37°C were labeled with Nile red, immobilized on an agarose pad, and then transferred to a thermostated microscope chamber at 20°C. We then performed TIR-FCS acquisitions on different cells for 1 h. As expected from a sudden temperature downshift, we observed first a reduction in diffusion coefficient (Fig. 3, *D* and *E*). Then, the diffusion coefficient progressively increased, likely caused by membrane adaptation, until reaching after ∼25–30 min the steady-state value previously measured for cells growing at 20°C (Fig. 3 *C*). This timescale of membrane fluidity adaptation is consistent with the significant membrane fatty acids remodeling within 30 min after a cold shock that was reported previously (46). The diffusion coefficients measured were fitted using the model of Eq. 5 accounting for membrane curvature, assuming a constant radius of 0.5 *μ*m and a cell length larger than 2.5 *μ*m (see Fig. S10). Manual measurements of cell width and length confirmed these hypotheses (Fig. S3).

### Temperature-induced changes in membrane fluidity in *S. aureus*

**Figure 4:**
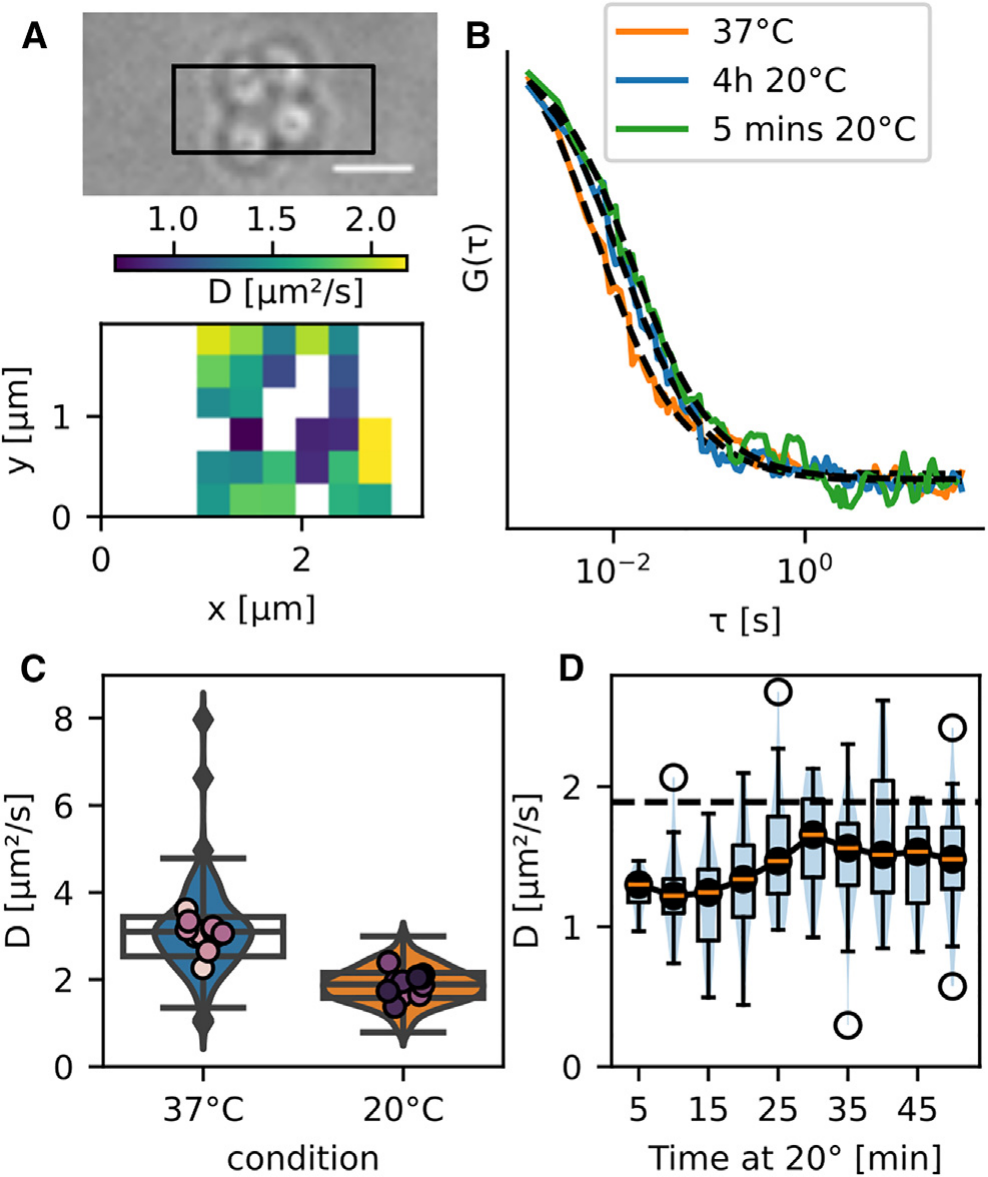
Adaptation of *S. aureus* membrane to cold temperature, measured with TIR-FCS. (*A*) Diffusion map of Nile red in *S. aureus* (*bottom*) and corresponding bright-field image (*top*, measured area in *black square*, scale bar, 2 *μ*m) at 20°C. (*B*) Normalized FCS curves obtained at 37°C (*orange*) and 5 min (*green*) and 4 h (*blue*) after transfer at 20°C. (*C*) Diffusion coefficient of Nile red in *S. aureus* cells grown and imaged at 37°C (*orange*) or 4 h after transfer at 20°C (*blue*). Dots: average of individual TIR-FCS acquisitions of one or more cells, two to three biological replicates, *n* > 150 FCS curves per condition. (*D*) Recovery of diffusion speed of Nile red upon transfer from 37 to 20°C, pooling measurements by tranches of 5 min, *n* = 3 biological replicates. Dotted black line: median diffusion coefficient of Nile red at 20°C from (*C*). To see this figure in color, go online.

We then set out to measure the membrane fluidity during cold shock of a different bacterium with a different geometry, the spherical Gram-positive pathogen *S. aureus*. Cold shock response has been thoroughly characterized in *B. subtilis* but is poorly understood in *S. aureus*. It was mainly found that, upon a drop in temperature, the membrane of *S. aureus* becomes enriched in carotenoids (63) and that cold shock stabilizes most of its RNA species (64). More recently, an analog of the *B. subtilis* two-component system DesK/DesR and transcription of *des* (encoding the fatty acid desaturase Des) responsible for temperature sensing was found in *S. aureus* (65), suggesting a similar response to cold shock in both bacteria. To confirm this, we conducted in *S. aureus* a series of experiments similar to the ones we performed in *B. subtilis*. Using TIR-FCS, we measured the diffusion speed of Nile red in the membrane of growing *S. aureus* cells at 37 and 20°C (Fig. 4 *A*). FCS curves were fitted with the standard TIR-FCS model (Eq. 1) and corrected for curvature using the simulated measurement bias calculated in Fig. 2 *B*. In practice, diffusion coefficients were divided by a factor of 2.1, corresponding to the bias induced by a sphere of radius 500 nm, corresponding to the radius of *S. aureus* we observed in our experiments (Fig. S5). For all conditions, we confirmed that cells kept growing on the slide before imaging, and we excluded cells that were not growing from the analysis. As expected, diffusion speed in steady-state measurements was higher at 37°C than at 20°C, like in *B. subtilis*, with, however, a slightly different ratio (Fig. 4 *C*; Table 2). We measured a similar diffusion coefficient of Nile red at 20°C for *B. subtilis* and *S. aureus* but a higher diffusion coefficient in *B. subtilis* at 37°C. When *S. aureus* cells were transferred from 37 to 20°C, we observed first a loss of fluidity due to the sudden change in temperature, followed by a recovery in fluidity to reach a plateau after ∼25–30 min, as also observed in *B. subtilis* (Fig. 4 *D*). The average diffusion coefficient of Nile red did not recover up to the 20°C steady-state value as in *B. subtilis* (Figs. 4 *D* and 3 *D*, respectively). We assumed that the recovery of membrane fluidity is nonetheless complete within 30 min and that the lower average diffusion coefficient we measured was caused by statistical variations between replicates (Fig. S6).

**Table 2:** Diffusion coefficients of Nile red in *S. aureus* at 37 and 20°C, and ratio of both diffusion coefficients

D_37°C_ (mean ± SD)	D_20°C_ (mean ± SD)	D_37°C_/D_20°C_
3.0 ± 0.3	1.9 ± 0.3	1.7 ± 0.4

## Discussion

In this paper, we demonstrate how TIR-FCS can be used to quantitatively measure membrane fluidity in bacteria, exemplified by the Gram-positive model organism *B. subtilis* and *S. aureus*. For this, we derived a new fit quality metric that greatly simplified data analysis. Using simulations validated by experiments, we estimated the measurement bias caused by the observation of a curved membrane of finite size in a TIRF field, and used this information to perform unbiased measurements of diffusion coefficients in the membrane of spherical and rod-shaped bacterial cells. We used this assay to measure membrane fluidity, reported by diffusion speed of membrane markers, in the membrane of *B. subtilis* and *S. aureus* at different temperatures, in steady state, and during membrane remodeling caused by a cold shock.

The fitting quality metric that we proposed is a fine addition to the collection of readily available quality metrics for FCS quality assessment. Its reliability across data sets with different signal levels might prove useful in future high-throughput FCS studies, whether camera based or not (66). Using TIR-FCS instead of conventional confocal FCS offered several advantages, including high-throughput measurements, excellent axial selectivity, allowing FCS measurements in single membranes and extraction of spatial information. Besides, the use of an unpolarized evanescent field with TIRF excitation allows excitation of fluorescent molecules irrespective of their dipole orientation. This is a major advantage when working with membrane markers such as Nile red or Di4 that intercalate within a membrane and keep a fixed orientation, which can be orthogonal to the polarization plane of, e.g., confocal excitation, and therefore lead to poor excitation quality and inhomogeneous excitation of observation area.

Simulating TIR-FCS in curved surfaces proved very useful to understand and measure the potential biases induced by bacterial membrane curvature, inhomogeneous excitation of the curved membrane in the evanescent field, and diffusion in a closed system of small size. The respective effect of each one these three parameters was not disentangled by our simulations. Further investigations would be required to quantify their individual contributions to the final measurement. Simple 2D simulations in finite-sized boxes could readily reveal that the small diffusion areas typical of bacterial membranes can lead to substantial biases in measured diffusion speed (Fig. S9). Care must be taken when accounting for membrane curvature with simulations to accurately simulate not only the observed geometry, but also the microscope itself as parameters such as the PSF size affect the final bias in diffusion coefficient (Fig. S10). Furthermore, we did not account for photobleaching in our simulations. This might affect the measurement bias caused by the closedness of the system: in a closed system, the same molecule is more likely to diffuse repeatedly through an observation area than in an open system. However, if there is significant bleaching, a molecule will likely diffuse only once through the observation area and then bleach. We do not expect this effect to significantly change the predictions of our simulations: bleaching was not simulated but occurred in BSLBs, and both systems exhibited a similar behavior (Fig. 2 *E*).

In this work, we have shown that in *B. subtilis* the diffusivity of two different membrane markers decreased similarly in response to a decrease in temperature, suggesting that membrane fluidity is the common determinant to their diffusivity. Claiming that our assay measures membrane fluidity requires, however, a couple of reasonable approximations. Membrane fluidity in the field of microbiology is usually referred to as a single parameter (the physical parameter governing diffusion speed in the plasma membrane). However, it is known that the fluidity in the physical sense affects the diffusivity of objects depending on their hydrophobic radius (67). The diffusivity of the membrane markers Nile red and Di4 is therefore a good proxy for the measurement of membrane fluidity experienced by molecules of comparable size (e.g., lipids). However, the fluidity experienced by larger molecules such as transmembrane or membrane-associated proteins is different. Relative differences in the diffusivity of transmembrane proteins and membrane markers between experimental conditions should nevertheless remain the same, provided that membrane fluidity is the main driver of their diffusivity. In *B. subtilis*, it was found using SPT that the diffusivity of a series of membrane proteins varied as expected with temperature-induced membrane fluidity variations and depended otherwise mostly on their number of transmembrane domains (68). However, interactions with nonmembrane cellular components may also affect the diffusivity of proteins, for instance, for proteins that are partly embedded in the cell wall through an intrinsically disordered region (69): in this case, cell wall properties are likely the main regulator of their diffusion speed. The slower diffusion speed of Nile red at 20°C relative to 37°C indicates that membrane proteins undergoing Brownian motion also diffuse more slowly at 20°C than at 37°C. This could be verified by performing a series of similar TIR-FCS experiments to measure the diffusivity of fluorescent protein fusions instead of membrane markers. Furthermore, we did not consider lateral membrane heterogeneities (named functional microdomains in bacteria (70), conceptually equivalent to lipid rafts (71) in eukaryotes) that are thought to recruit certain proteins and lipids in areas of higher molecular order and therefore lower mobility. The diffusivity that we measured in this study likely represents an average of the diffusivity in all domains of the membrane.

Our measurements were performed in Gram-positive bacteria, which lack an outer membrane. More care will need to be taken to perform TIR-FCS experiments on membrane markers in Gram-negative bacteria. First, the fluorescent marker used should label only the membrane of interest, either the inner or the outer membrane. Second, TIR-FCS experiments will be precluded in experimental conditions where the topology of the membrane is affected, such as in presence of certain antibiotics (28,72), as changes in membrane topology significantly bias diffusion coefficient estimation with FCS (73).

Our *B. subtilis* TIR-FCS data confirm the effect of a cold shock on membrane fluidity previously reported using fatty acid analysis and environment-sensitive dyes (47,74). Upon cold shock, membrane fluidity initially decreases but rapidly increases again due to a modification of plasma membrane composition that involves an increase in unsaturated and branched-chain fatty acids in the membrane (47). However, it remained unknown to what extent membrane fluidity recovered. Fatty acid profiles only provided qualitative information and conflicting results were obtained with environment-sensitive probes (74). DPH anisotropy suggested an incomplete recovery of *B. subtilis* membrane fluidity while fluorescence lifetime measurements of the same probe indicated an identical fluidity at 20 and 37°C (74). Our results allow to settle this debate unambiguously: after a cold shock, membrane fluidity does not completely recover to the precold shock value in *B. subtilis*, it recovers to the steady-state fluidity at the temperature to which cells were transferred.

We have demonstrated the ability of our assay to measure membrane fluidity in coccoid bacteria as well by studying the diffusivity of Nile red in the membrane of *S. aureus*. Little was known about the adaptation of the plasma membrane of *S. aureus* to a cold shock, except that the membrane gets enriched in carotenoids (63) and that a thermosensor resembling that of *B. subtilis* (the DesK/DesR two-component system) is expected to regulate plasma membrane fluidity (65). Our work revealed a similar recovery time after cold shock in both bacteria, both occurring within ∼30 min. As in *B. subtilis*, the fluidity of *S. aureus* recovers to its steady-state value at 20°C, which is lower than its value at 37°C. Our measurements also revealed that membrane fluidity is lower in *S. aureus* than in *B. subtilis* at 37°C and similar in both species at 20°C. Therefore, the quantitative and purely physical measure provided by our assay also allows for interspecies comparison.

Why do *B. subtilis* and *S. aureus* not maintain the same membrane fluidity across temperatures? An explanation might be that membrane fluidity homeostasis occurs only below a certain critical temperature, below which the plasma membrane transitions to a deadly gel-like phase. This is supported by previous findings that activation of the DesK thermosensor in *B. subtilis* occurs only below ∼30°C (75,76). In this scenario, membrane fluidity would be regulated only at low temperatures to prevent fluidity from dropping below a certain critical threshold. Temperature increases above 30°C would not lead to changes in membrane composition, leading to an increase in membrane fluidity caused by thermodynamics.

In conclusion, our TIR-FCS assay opens up exciting perspectives in the field of microbiology. The unprecedented ability to directly quantify membrane fluidity will shed a new light on the biophysics of bacterial membranes and might help to understand key cellular processes such as membrane remodeling upon viral infection or changes in nutriment availability, as well as the mode of action of membrane-targeting antibiotics. This newly acquired capability to quantify membrane fluidity will also enhance our understanding of the fundamental role of membrane fluidity in bacterial physiology.

## Data and code availability

Research data are available on Zenodo: https://doi.org/10.5281/zenodo.11236214. Code and manual of the FCS analysis software developed for this project and used throughout this paper can be found at https://github.com/aurelien-barbotin/pyimfcs. Code for simulations can be found at https://github.com/aurelien-barbotin/geomdsim.

## Author contributions

A.B., C.B., E.S., and R.C.-L. designed the project and wrote the manuscript. A.B. performed the experiment, analyzed data, and wrote the code.

## Supporting material

Document S1. Figures S1–S10

Document S2. Article plus supporting material

## Footnotes

Supporting material can be found online at https://doi.org/10.1016/j.bpj.2024.06.012.
