# Supplementary material for: Quantification of membrane fluidity in bacteria using TIR-FCS
Source: Biophys J. 2024 Jun 13;123(16):2484–95. doi: 10.1016/j.bpj.2024.06.012 (PMC11365102; doi:10.1016/j.bpj.2024.06.012)
Supplement: Document S1. Figures S1–S10 [file mmc1.pdf]

Biophysical Journal, Volume *123*

Supplemental information

Quantification of membrane fluidity in bacteria using TIR-FCS

Aurélien Barbotin, Cyrille Billaudeau, Erdinc Sezgin, and Rut Carballido-López

Quantification of membrane fluidity in bacteria using TIR-FCS: Supporting material

Aurélien Barbotin[1, *], Cyrille Billaudeau[1] ,Erdinc Sezgin[2], Rut Carballido-López[1, *]

1 Université Paris-Saclay, INRAE, AgroParisTech, Micalis Institute, 78350, Jouy-en-Josas, France.
2 Science for Life Laboratory, Department of Women’s and Children’s Health, Karolinska Institutet, 17165 Solna, Sweden
* correspondance: aurelien.barbotin@inrae.fr, rut.carballido-lopez@inrae.fr

Live bacteria

Influence of label concentration on FCS outcome

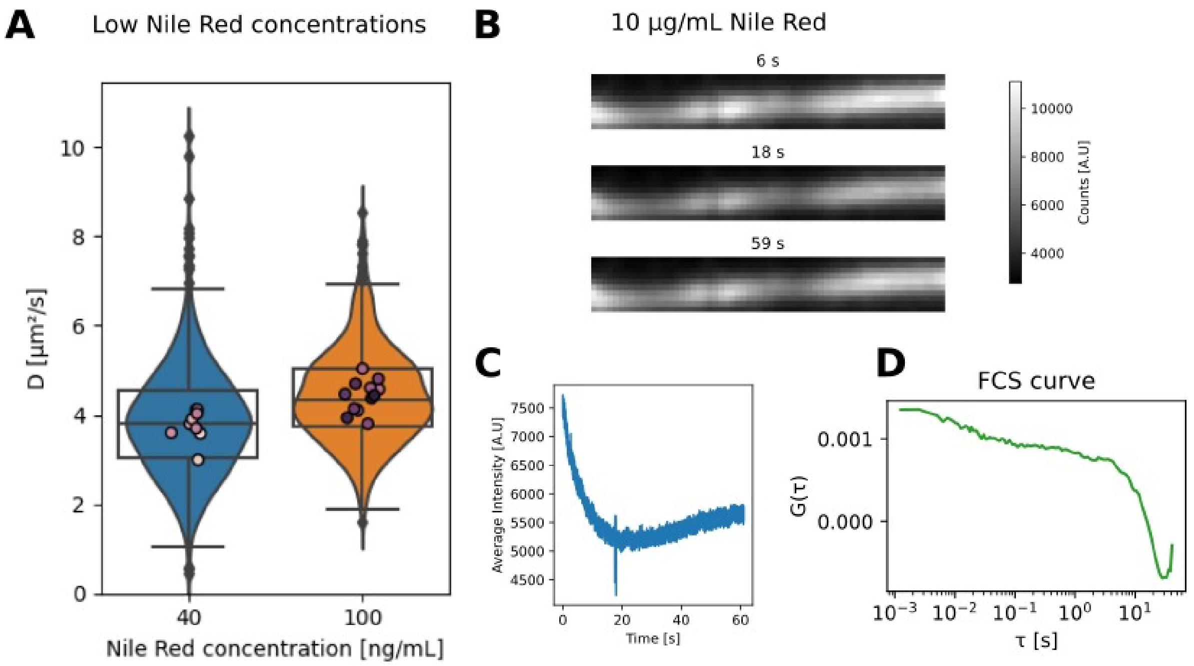

Figure S1 Influence of Nile Red concentration on diffusion coefficient measurement with TIR-FCS in B. subtilis at 37°C. (A) Diffusion coefficient of Nile Red in the membrane of B. subtilis labeled with two different Nile Red concentrations. (B) Average of 1000 frames of a TIR-FCS acquisition in B. subtilis labeled with 10 µg/mL Nile Red concentration, after 6 (top), 18 (middle) and 59 (bottom) seconds of acquisition. (C) Time-dependent intensity averaged across all pixels of the TIR-FCS acquisition shown in (B). (D) representative FCS curve obtained from the acquisition shown in (B)

We tested whether the concentration of membrane marker influenced the outcome of TIR-FCS experiments. For this, we performed TIR-FCS measurements of the diffusion coefficient of Nile Red in the membrane of exponentially-growing *B. subtilis* cells at 37°C, labelled with either a final concentration of 40 or 100 ng/mL of Nile Red (Fig. S1A). We found similar diffusion coefficients, suggesting that at low concentrations, labeling concentration does not affect the outcome of FCS measurement, as expected (7). A small (~10%) difference between the two concentrations was most likely due to variations in medium properties, marker aliquot or simply statistical variations. At higher concentrations, however, we noticed that during FCS acquisitions, fluorescence intensity

first decreased as expected because of photobleaching but then quickly increased again (Fig. S1B-C). This in turns created artefacts in FCS curves that prevented reliable diffusion coefficient estimation (Fig. S1D). This unexpected increase in fluorescence intensity was likely caused by a membrane remodelling due to phototoxicity, which increases with label concentration.

Diffusion coefficient of Nile Red in the membrane of *B. subtilis* as a function of time spent on agarose pad.

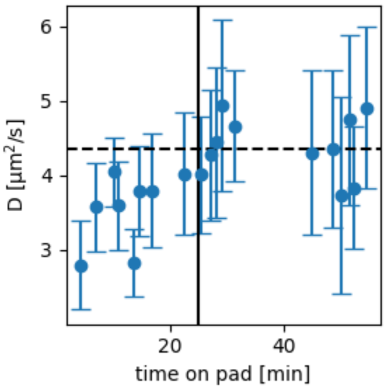

Figure S2: Diffusion coefficient as a function of time spent on an agarose pad at 37°C in growing B. subtilis. Steady-state fluidity is reached after approximately 25 mins (vertical black line), after which imaging can start.

B. subtilis morphology in different conditions

Having found that the width and length of bacterial cells bias diffusion coefficient measurements, we verified whether the morphology of *B. subtilis* cells changed between the different experimental conditions investigated here. For this, we acquired epifluorescence images for each of these conditions (Fig. S3A-B) and measured cell length and width manually using ImageJ. Cell length was determined by drawing a line between the two poles of each cell and measuring the length of this line. We found that the average cell length was identical at 37°C and immediately after cold shock, decreased after 5hours at 20°C (Fig. S3C), but not to a point where cell length biased FCS measurements.

Cell width was measured by plotting the intensity profile alongside a line orthogonal to the cell long axis and measuring the peak-to-peak distance. This method led to an underestimation of the real cell width due to off-axis fluorescence emitted by the top and bottom part of the membrane, hence the relative difference with the well-known diameter of *B. subtilis* of 0.9-1μm (3-4). It revealed however that as expected cell width did not change significantly between experimental conditions and thus that we could apply the same correction factor accounting for membrane curvature to measurements (Fig. S3D). Cell width remained constant during cold shock (Fig. S3F) and cell length remained well above the 2.5 μm threshold leading to bias in diffusion coefficient (Fig. S10A). Panels C and D of Fig. S3 were generated using supplementary ref 1.

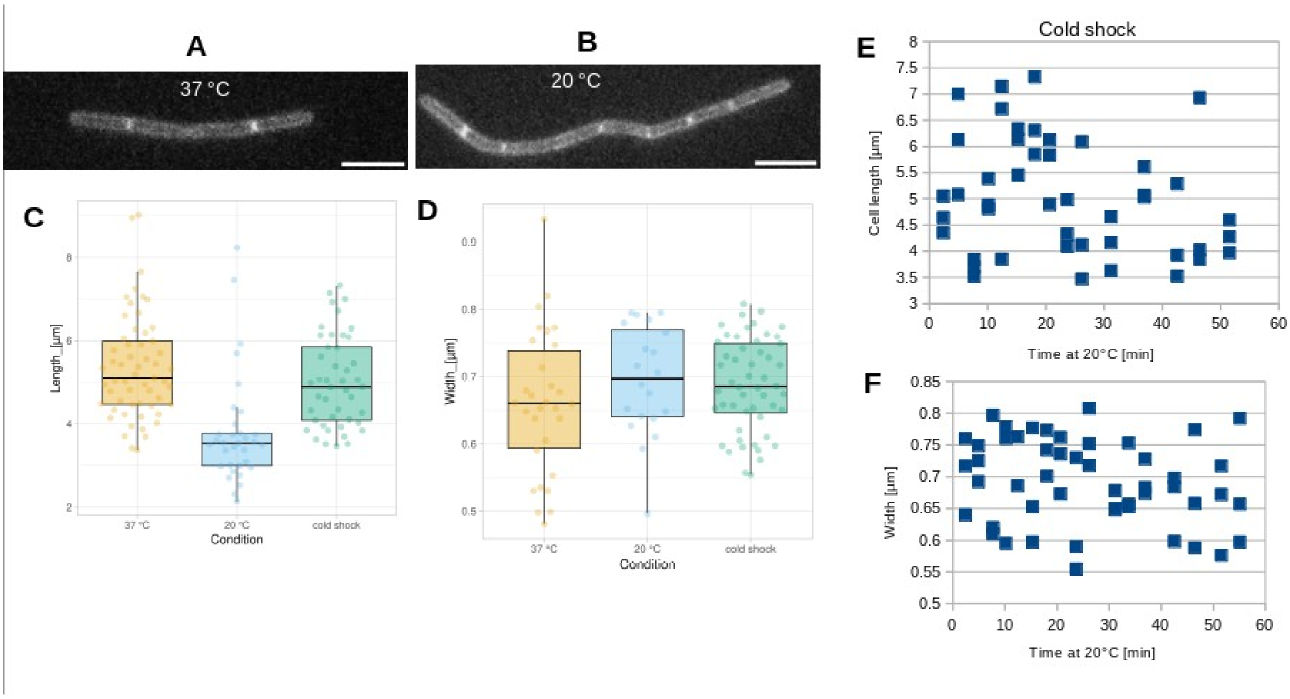

Figure S3: Morphology of B. subtilis at different temperatures. (A-B) epifluorescence images of B. subtilis in exponential phase, labeled with Nile Red, at 37°C (A) and 20 °C (B). Scalebars: 5 µm. (C-D) Length (C) and width (D) of B. subtilis measured from epifluorescence images, in exponential phase at 37°C, 20°C, or during cold shock. (E-F) Scatterplots of cell length (E) and width (F) with time at 20°C immediately after cold shock.

Impact of FCS measurement on doubling time in *B. subtilis*

Using bright-field timelapses, we verified both cell fitness and the impact of phototoxicity on cell growth. For this, we measured the growth rate of cells used in Fig. 3C, at 37°C. We acquired for each chain of cells 3 bright-field images (Fig. S4A), one at least 3 mins before the beginning of FCS acquisition, one immediately after the FCS acquisition and one at least 3 mins after FCS acquisition. We measured the length of the cell chain in each bright-field image and calculated doubling times between pairs of frames following the equation (under the assumption of constant cell width as is the case in *B. subtilis*):

$$T_{double} = \Delta t \ln(2) / \ln(l_2 / l_1) \qquad 1$$

Where T_{double} is the doubling time, Δt is the time between frames 1 and 2, l_1

and l_2 are the lengths of the cell chain in frames 1 and 2. Cells which doubling was more than twice higher than the nominal doubling time (~20 mins) were considered not exponentially-growing and therefore excluded from the analysis. Comparing pairwise doubling times before and after FCS (Fig. S4B), we found that cells kept growing after FCS, yet at a slightly slower rate, suggesting low photoxicity effects.

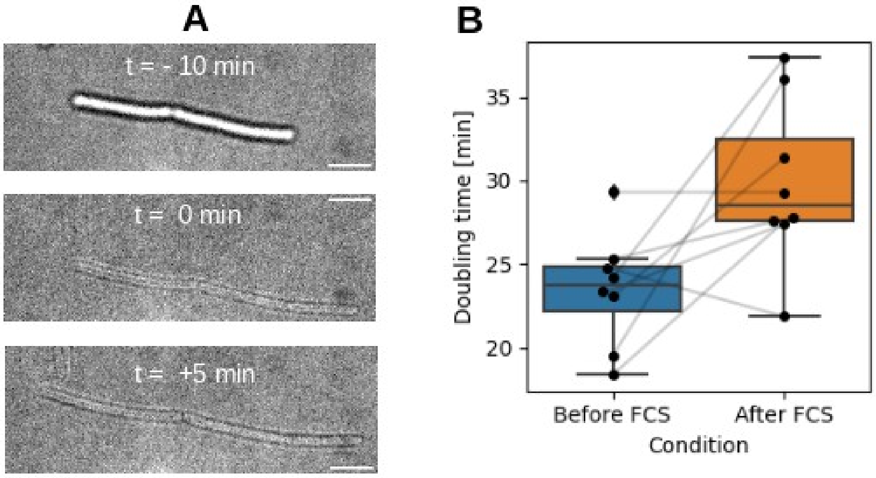

Figure S4: Impact of FCS measurements on the growth rate of Nile Red-labeled B. subtilis cells. (A): bright-field images of growing cells acquired before (top), immediately after (middle) and after (bottom) FCS acquisition. Scale bars: 5 µm. (B) Doubling times calculated from cell elongation, before and after FCS acquisition. Black dots: single doubling times measurements, gray lines link doubling times of the same cell chain.

S. aureus morphology in different conditions

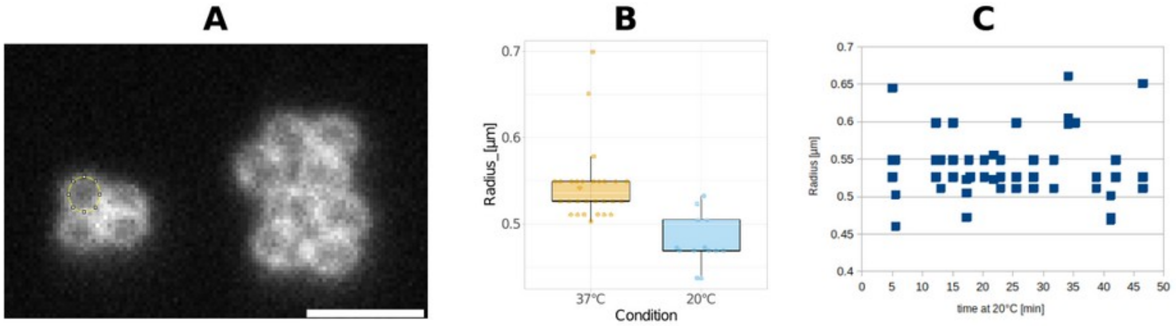

Figure S5 Measuring cell radius of S. aureus at different temperatures. (A) Cell area is measured by manually fitting an ellipsoid (yellow) to epifluorescence images of S. aureus cells labeled with Nile Red. Scale bar: 3 µm. Cells diameter is extracted from area either in a steady-state (A) or during a cold shock (B).

Membrane fluidity in bacteria

We corrected the bias induced by the curvature of S. aureus cells using simulations, assuming that S. aureus cells were spheres of diameter 500 nm. We verified this experimentally in all our experimental conditions using images of S. aureus cells labeled with Nile Red. The area *A* of circular cells was extracted using ImageJ by manually fitting an ellipsoid (Fig. S5A) to the membrane of cells, then their radius *R* was estimated using the formula $R=\sqrt{(A/\pi)}$. Our results confirmed that the radius of S. aureus cells was indeed ~500 nm in all our imaging conditions.

Individual cold shock replicates in staphylococcus aureus

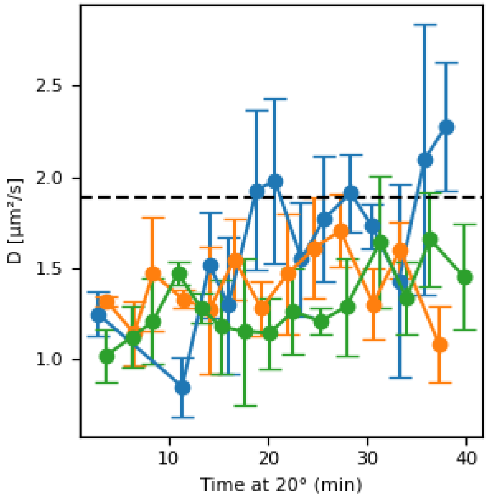

Figure S6: Diffusion coefficient of Nile Red in staphylococcus aureus after transfer at 20°C. Median +/- std of individual acquisitions in the 3 different replicates. Dotted black line: steady-state diffusion coefficient at 20°C.

Detailed implementation of FCS data processing

Intensity threshold

In order to avoid biasing diffusion measurements, we needed to exclude TIR-FCS measurements that were too far from the point of contact between the bacterial cell and the coverslip. An efficient way of doing this consisted in removing pixels with an average intensity below a given threshold, as the excitation of the TIRF field decreases with increased distance to the cell centre. To find an appropriate value for this intensity threshold, we plotted a 2D histogram of intensity (normalised with 98th percentile) and diffusion coefficient in 6 acquisitions of exponentially-growing *B. subtilis* labeled with Nile Red at 20°C. We set the threshold to 0.8 so that there was no correlation between diffusion coefficient and intensity (Fig. S7). We kept the same threshold for *S. aureus* cells. In S. aureus, we applied this intensity threshold not to the whole image but to individual cells, in order to avoid biasing results when one or more cells was brighter than the others in a field of view. The outlines of individual cells were found automatically using a watershed algorithm.

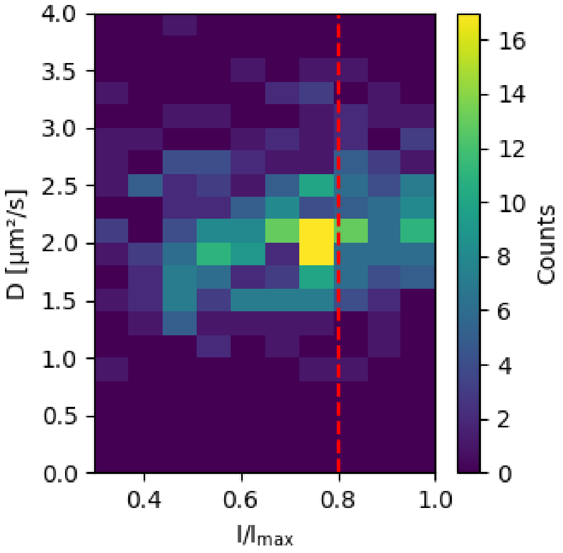

Figure S7: Determination of intensity threshold for unbiased diffusion measurement. Correlation between relative pixel intensity (x axis) and measured diffusion coefficient (y axis) visualised as a 2-dimensional histogram in 6 acquisitions of exponentially-growing B. subtilis at 20°C. Vertical dotted red line: selected threshold

Bleaching correction and FCS fitting

Bleaching correction was performed using a double exponential fit of the decaying intensity. Intensity timetraces were downsampled 500 times to speed up computations. The resulting traces were fitted with the function:

$$\hat{I}(t)=f_0\left[(1-b)\exp(-t/\tau_1)+b\exp(-t/\tau_2)\right]+c \qquad 2$$

The original intensity timetrace was then corrected as described in ref (2):

$$I_c(t)=\frac{I(t)}{\sqrt{\hat{I}(t)/\hat{I}(0)}}+\hat{I}(0)\left(1-\sqrt{\hat{I}(t)/\hat{I}(0)}\right) \qquad 3$$

The error function in Eq. 1 is defined as :

$$erf(x)=\frac{2}{\sqrt{\pi}}\int_0^x \exp(-t^2)dt \qquad 4$$

Simulations

On a sphere: First a set of points representing individual fluorescent emitters were distributed randomly on a sphere, as described in (5). The position of each point was described in spherical coordinates. Its azimuthal (θ) and polar (φ) angles were randomly generated using the following equation:

$$\theta = 2\pi u$$
$$\phi = \cos^{-1}(2v - 1) \quad 5$$

where u and v are drawn from uniform random variables with bounds [0,1[. Trajectories were then converted to cartesian coordinates. At a given time t, the vector position $\vec{r}(t) = [x(t), y(t), z(t)]$ of a point was then updated as follows, as discussed in reference (6):

$$\vec{r}(t+1) = \frac{R}{\|\vec{r}(t) + \vec{r}(t) \wedge \vec{b}(t)\|}(\vec{r}(t) + \vec{r}(t) \wedge \vec{b}(t)) \quad 6$$

where R is the radius of the sphere and $\vec{b}(t) = [u_x(t), u_y(t), u_z(t)]$ is a three-dimensional random vector drawn from a normal distribution, with each component having a standard deviation of $\sqrt{2Dt}/R$, with D the diffusion coefficient. The normalisation factor $\frac{R}{\|\vec{r}(t) + \vec{r}(t) \wedge \vec{b}(t)\|}$ is necessary to keep the vector $\vec{r}(t+1)$ on the surface, as the vector $\vec{r}(t) \wedge \vec{b}(t)$ is tangential to the curved surface and therefore $\vec{r}(t) + \vec{r}(t) \wedge \vec{b}(t)$ is not on the surface (Fig. S8A). Under the conditions that the angle between $\vec{r}(t)$ and $\vec{r}(t+1)$ is small, $\|\vec{r}(t+1) - \vec{r}(t)\| = \vec{r}(t) \wedge \vec{b}(t)$ and the simulation of brownian motion is accurate.

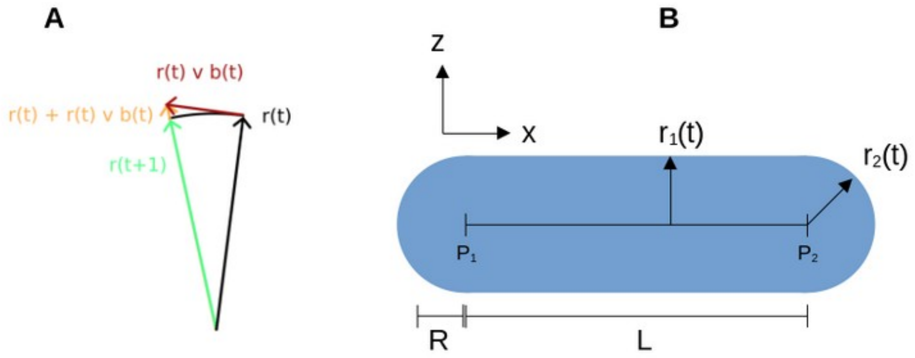

Figure S8: Sketch of the simulation of a Wiener process on curved surfaces. (A): iteration of the Wiener process. B: Sketch of rod-shape simulation.

On a rod: the simulation process is very similar. The rod is represented as a cylinder of length L and radius R with two half-spherical parts or radius R at its end (Fig. S8B), oriented along the x-axis. The initial distribution of points is done in two steps: a fraction of the total number of the points is distributed on a sphere, while the rest of the points are distributed on a cylinder. The relative fraction of points on the sphere and the cylinder is determined from the relative areas of the spherical and cylindrical parts of the rod. Points drawn on the sphere with a negative x coordinate are moved along the x axis by a distance -L/2, the others by a distance +L/2. The vector position $\vec{r}(t)$ of each point is then iteratively updated following Eq. 6 as in the previous section, except that in this case the vector $\vec{r}(t)$ describes the distance to the medial axis (segment [P_1P_2] in Fig.

S8B) of the rod and not to the centre of the sphere. Fig. S8B illustrates the two different configurations ($\vec{r}_1(t)$ and $\vec{r}_2(t)$) for the vector $\vec{r}(t)$.

Parameters used in the simulations of Fig. 2 are listed in the following table:

**Frame rate (kHz)**	**D [μm²/s]**	**# frames**	**Parts. density [parts/μm²]**	**Length [μm] (rod only)**	**Pixel size [μm]**	**σ_{psf} [μm]**	**δz_{TIRF} [μm]**	**Brightness (Hz)**
1	1	50000	1.6	3	0.08	0.19	0.1	20000

Particle density was set to the constant value of 1.6 particle/μm², except in smallest simulations for which it was increased to contain at least 10 particles. TIRF penetration depth δz was defined as:

$$I_{TIRF}(z)=I_0 \exp(-z/\delta z) \quad 7$$

Where I(z) is the depth-dependent TIRF excitation field. To speed up calculations, all particles above 4δz were considered to have a brightness equal to zero and were discarded from the analysis. Analysis was performed with 4x4 binning to an observation area of 320 nm, similar to the one we used in our experiments. An intensity threshold set to 80% of the maximum intensity was also used to analyse simulations as we used in real experiments. Each simulation was performed 9 times. The lateral position of the simulated bacterium was different for each of the 9 simulations to avoid a potential bias.

Influence of size of closedness of the system:

In order to understand if the closedness of the simulated systems described above and in Fig. 2 could lead to a bias in diffusion coefficient estimation with imFCS, we simulated a simple system of 2-dimensional Brownian motion in a homogeneous illumination field. Molecules leaving the system on one edge were reintroduced at the corresponding position on the opposite edge (Fig. S9A). When the box became very small, we could observe that FCS curves shifted towards shorter lag times and became distorted (Fig. S9B). Fitting curves for different box sizes to extract diffusion coefficients confirmed that smaller box sizes, of areas in the order of magnitude of bacterial membrane areas, indeed induced a bias in diffusion coefficient estimation (Fig. S9C). It is therefore very likely that part of the measurement biases in Fig. 2 were caused by an effect of the small size of the systems observed.

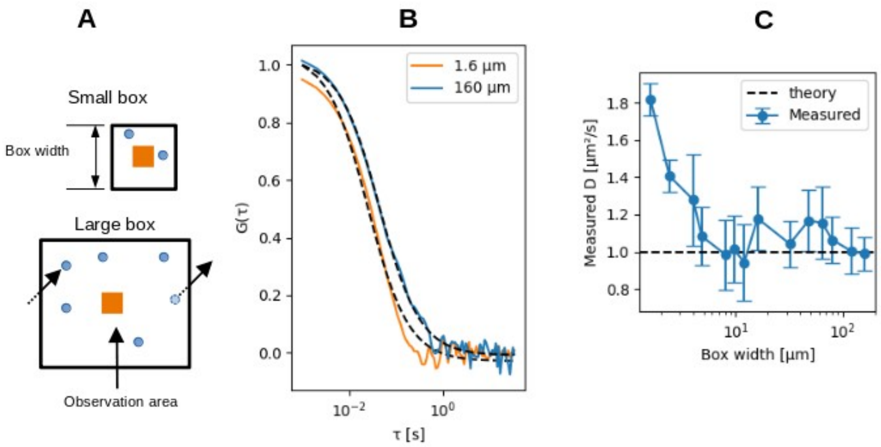

Figure S9: TIR-FCS simulations in a closed box of varying size. (A) sketch of the simulated system with box (black) containing moving particles (blue) and a smaller observation area (centre, orange square) where TIR-FCS is simulated. Top: small box, bottom: large box. A molecule leaving on one side and reentering on the other side is shown with dashed arrows. (B) representative FCS curves obtained when a small (1.6 µm width, orange) and large (160 µm width, blue) simulation box are used. (C) Measured diffusion coefficients in simulated TIR-FCS experiment as a function of box size.

Influence of rod length and PSF size:

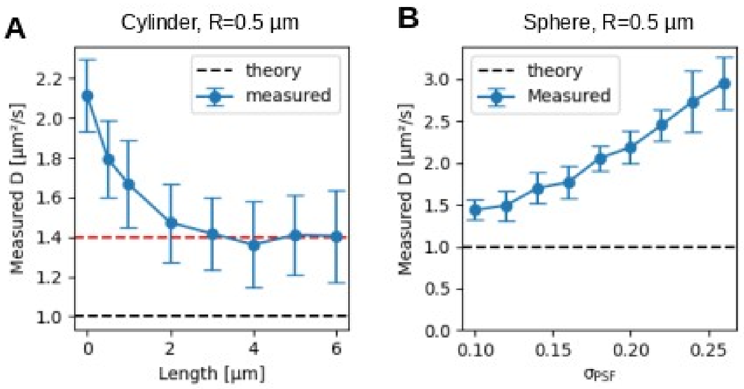

Figure S10: Simulation of the influence of physical parameters on measured diffusion coefficient. (A) Measured diffusion coefficient with length of rod-shape, for a radius of 0.5 µm. Red dashed line: bias for lengths>2.5 µm. (B) Influence of PSF size (parameter σ in Eq. 1-2) on measured diffusion coefficient, on a sphere of radius 0.5 µm.

Supporting References :

1. Lord, S. J.; Velle, K. B.; Mullins, R. D.; Fritz-Laylin, L. K. SuperPlots: Communicating Reproducibility and Variability in Cell Biology. *Journal of Cell Biology* **2020**, *219* (6), e202001064. https://doi.org/10.1083/jcb.202001064.

2. Ries, J., S. Chiantia, and P. Schwille. 2009. Accurate Determination of Membrane Dynamics with Line-Scan FCS. *Biophysical Journal*. 96:1999–2008.

3. Errington J, Aart LTV. 2020. Microbe Profile: *Bacillus subtilis*: model organism for cellular development, and industrial workhorse. Microbiology (Reading). May;166(5):425-427.

4. Juillot, D., C. Cornilleau, N. Deboosere, C. Billaudeau, P. Evouna-Mengue, V. Lejard, P. Brodin, R. Carballido-López, and A. Chastanet. 2021. A High-Content Microscopy Screening Identifies New Genes Involved in Cell Width Control in Bacillus subtilis. 6.

5. https://www.bogotobogo.com/Algorithms/uniform_distribution_sphere.php

6. https://math.stackexchange.com/questions/3725288/infinitesimal-generator-of-the-brownian-motion-on-a-sphere

7. Schneider, F., P. Hernandez-Varas, B. Christoffer Lagerholm, D. Shrestha, E. Sezgin, M. Julia Roberti, G. Ossato, F. Hecht, C. Eggeling, and I. Urbančič. 2020. High photon count rates improve the quality of super-resolution fluorescence fluctuation spectroscopy. *J. Phys. D: Appl. Phys.* 53:164003.
