# Supplementary material for: Quantification of membrane fluidity in bacteria using TIR-FCS
Source: Biophys J. 2024 Jun 13;123(16):2484–95. doi: 10.1016/j.bpj.2024.06.012 (PMC11365102; doi:10.1016/j.bpj.2024.06.012)
Supplement: Document S2. Article plus supporting material [file mmc2.pdf]

Biophysical Journal
Article

Biophysical Society

Quantification of membrane fluidity in bacteria using TIR-FCS

Aurélien Barbotin,[1,*] Cyrille Billaudeau,[1] Erdinc Sezgin,[2] and Rut Carballido-López[1,*]
[1]Université Paris-Saclay, INRAE, AgroParisTech, Micalis Institute, Jouy-en-Josas, France and [2]Science for Life Laboratory, Department of Women's and Children's Health, Karolinska Institutet, Solna, Sweden

ABSTRACT Plasma membrane fluidity is an important phenotypic feature that regulates the diffusion, function, and folding of transmembrane and membrane-associated proteins. In bacterial cells, variations in membrane fluidity are known to affect respiration, transport, and antibiotic resistance. Membrane fluidity must therefore be tightly regulated to adapt to environmental variations and stresses such as temperature fluctuations or osmotic shocks. Quantitative investigation of bacterial membrane fluidity has been, however, limited due to the lack of available tools, primarily due to the small size and membrane curvature of bacteria that preclude most conventional analysis methods used in eukaryotes. Here, we develop an assay based on total internal reflection-fluorescence correlation spectroscopy (TIR-FCS) to directly measure membrane fluidity in live bacteria via the diffusivity of fluorescent membrane markers. With simulations validated by experiments, we could determine how the small size, high curvature, and geometry of bacteria affect diffusion measurements and correct subsequent measurements for unbiased diffusion coefficient estimation. We used this assay to quantify the fluidity of the cytoplasmic membranes of the Gram-positive bacteria *Bacillus subtilis* (rod-shaped) and *Staphylococcus aureus* (coccus) at high (37°C) and low (20°C) temperatures in a steady state and in response to a cold shock, caused by a shift from high to low temperature. The steady-state fluidity was lower at 20°C than at 37°C, yet differed between *B. subtilis* and *S. aureus* at 37°C. Upon cold shock, the membrane fluidity decreased further below the steady-state fluidity at 20°C and recovered within 30 min in both bacterial species. Our minimally invasive assay opens up exciting perspectives for the study of a wide range of phenomena affecting the bacterial membrane, from disruption by chemicals or antibiotics to viral infection or change in nutrient availability.

SIGNIFICANCE Using fluorescence correlation spectroscopy (FCS) with total internal reflection fluorescence (TIRF) illumination, we measured the diffusion speed of fluorescent membrane markers as a readout for membrane fluidity of growing *B. subtilis* and *S. aureus* cells. Monitoring the effect of cold shock provided unique information about the dynamics of the plasma membrane of these two bacterial species. The unprecedented capability of TIR-FCS to quantify the fluidity of a single membrane in living bacteria opens the door to a whole set of new studies that will shed light on the mechanisms underlying bacterial membrane homeostasis and its interactions with the environment.

INTRODUCTION

The plasma membrane, also called the cell or cytoplasmic membrane, is a component of every living cell. It is a fluid mixture of lipids and proteins that separates the intracellular and extracellular spaces. The fluidity (inverse of the viscosity) of the plasma membrane is the physical parameter that defines how fast a given element can diffuse within the membrane at a given temperature. Thus, membrane fluidity is of utmost interest for protein diffusion and biomolecular interactions (1,2). Membrane fluidity also affects protein folding (3–5). In bacteria, membrane fluidity is known to vary in response to chemical (6), biochemical (7,8), and osmotic (9,10) stresses. Membrane fluidity has been shown to be critical in both Gram-negative bacteria (for respiration in *Escherichia coli* (11) and multidrug transport in *Methylobacterium extorquens* (12)) and Gram-positive bacteria (e.g.,., resistance to antibiotics in *Staphylococcus aureus* (13), adaptation to extreme growth conditions in *Staphylococcus haemolyticus* (14), and response to antimicrobial peptides in *Bacillus subtilis* (15)). Another hint of the importance of membrane fluidity in bacterial cells lies in the widespread existence of control mechanisms that maintain it by

Submitted March 1, 2024, and accepted for publication June 10, 2024.

*Correspondence: aurelien.barbotin@inrae.fr or rut.carballido-lopez@inrae.fr

Editor: Gerhard Schutz.

https://doi.org/10.1016/j.bpj.2024.06.012

© 2024 Biophysical Society.

This is an open access article under the CC BY license (http://creativecommons.org/licenses/by/4.0/).

modifying lipid and protein composition (16,17). In particular, the fatty acid composition of phospholipids—the main class of lipids of the plasma membrane—is modified in response to changes in temperature to modulate steric constraints and thereby lipid packing (18). Furthermore, proteins such as flotillins (19,20) and MreB (21) are also thought to play a role in membrane fluidity in *B. subtilis*. In the case of extremophiles, the generation of exopolymers (22), cryoprotectants, and antifreeze proteins (23) are used to protect the bacterial membrane against changes in temperature.

Existing methods to characterize membrane fluidity in live and synthetic membranes include electron spin resonance (24,25) and NMR spectroscopy (19), membrane fatty acid analysis (9,13,24), fluorescence assays using environment-sensitive probes such as diphenylhexatriene (DPH), or ratiometric probes such as Laurdan (6), and the measurement of the diffusion speed of a fluorescent tracer using either single-particle tracking (SPT) (26), fluorescence recovery after photobleaching (27), or fluorescence correlation spectroscopy (FCS) (28).

In microbiology, the most frequently used techniques are fatty acid analysis and DPH anisotropy or ratiometric imaging with environment-sensitive probes (typically Laurdan). Fatty acids analysis partially informs on the membrane composition but is an indirect readout of fluidity. It gives multidimensional results (relative proportions of different fatty acids with branching and (poly)unsaturation), which can be challenging to directly associate with a change in membrane fluidity, and can in the best case only provide qualitative comparisons of the resulting fluidity. Environment-sensitive fluorescent probes can give useful insights but can only measure relative differences in membrane fluidity. Many probes exist that are sensitive to different parameters of the membrane (29,30) and their behavior can be biased by unforeseen interactions (31).

FCS has occasionally been used in a few instances in bacterial membranes, to study protein diffusion (32–34), RNA concentration (35), assembly of protein complexes (36), or membrane dynamics in response to antibiotic treatment (28). These studies were all performed using confocal microscopy, the axial resolution of which (~600 nm) is not well suited for measurements in bacteria. The axial resolution of confocal microscopes is comparable with the diameter of most studied bacterial cells (500 nm–1 μm), and this results in problems such as such as having both top and bottom membranes in focus at once or excessive background from out-of-focus membranes. These limitations can be overcome either by using super-resolution microscopy (37) or more simply by using total internal reflection fluorescence (TIRF) microscopy. TIRF microscopy significantly improves the axial resolution of a microscope by illumination with an evanescent field that usually significantly decays within a range of 100 nm at the interface between the coverslip and the sample, which makes it ideally suited for the investigation of events at the cell surface.

Total internal reflection-fluorescence correlation spectroscopy (TIR-FCS) (38,39) was previously used to study molecular dynamics in eukaryotic cells (40,41) and to measure the fluidity of flat synthetic membranes (42,43). However, TIR-FCS was not previously applied to bacteria despite TIRF having become common for the study of molecular dynamics in the bacterial membrane (44). TIR-FCS offers several advantages over confocal FCS, besides the unrivalled axial selectivity of TIR illumination. First, camera-based TIR-FCS also offers massive parallelization of measurements: hundreds of FCS curves can be acquired simultaneously instead of a single one on a confocal microscope. Second, TIR-FCS can easily generate diffusion maps and therefore retrieve spatial information. Finally, TIR-FCS enables, by resampling intensity fluctuations in space after acquisition, the measurement of diffusion speeds at different spatial scales (spot-variation FCS (45)).

Here, we extend the scope of application of TIR-FCS to measure membrane fluidity in live bacterial cells with different morphologies, exemplified by the Gram-positive leading model organism *B. subtilis* and the pathogen *S. aureus*, which are respectively rod-shaped and spherical. To simplify data analysis and fully use the high-throughput capability of imaging FCS, we developed a new FCS quality metric to automatically discard artifactual curves. Using simulations validated by experiments in synthetic samples, we measured the bias induced by the small size and curvature of bacterial membranes on TIR-FCS measurements. We demonstrated the validity of our assay by studying the well-known response of the *B. subtilis* plasma membrane to a cold shock (46,47) and the much less-studied cold shock response of *S. aureus*. Diffusion measurements of the membrane markers Nile red and Di4-ANEPPS confirmed the previous knowledge about cold shock recovery in *B. subtilis* and provided unprecedented insights of bacterial membrane dynamics at different temperatures in both *B. subtilis* and *S. aureus*.

MATERIALS AND METHODS

FCS setup

TIR-FCS acquisitions were performed on a Zeiss Elyra PS1 microscope equipped with a 100×/1.46 NA Apochromat oil immersion objective. A typical FCS acquisition consisted of 50,000 frames, on a field of view of 128 × 10 pixels with a pixel size of 160 nm in the object plane and a frame acquisition time of 1.26 ms, the maximum achievable with our camera. Stable focus was ensured using Definite Focus. Detection was performed using an emCCD camera (Andor iXon 897), using maximum preamplification (5×) and electron-multiplying gains (300×) settings. Laser excitation at 561 nm was set, unless specified otherwise, to 5% of the maximum excitation power in *B. subtilis* and synthetic samples and 10% in *S. aureus*. 5% of the maximum excitation corresponded to a power of 460 μW in epifluorescence mode measured in the focal plane of the objective. The excitation area was of approximately 80 × 80 μm size, leading to an estimated power density of ~70 nW/μm^2.

Data processing and fitting

Pixels from each image stack were numerically binned 2 by 2, unless specified otherwise. The first 1500 frames of every acquisition were discarded as an occasional loss of focus could lead to artifactual intensity fluctuations in these frames. Intensity timetraces at each binned pixel were corrected for bleaching using a double exponential fit (48). Intensity traces at each binned pixel were correlated using a python implementation of the multipletau algorithm (49). FCS curves were fitted using the standard two-dimensional (2D) imaging FCS model (50) (except in *B. subtilis* cells where the fitting model is described in impact of membrane curvature, Eq. 5):

$$\begin{aligned} g_{xy}(\tau) &= \frac{1}{N}\left(\frac{1}{\sqrt{\pi}\mu}\left(exp(-\mu^2) - 1\right) + erf(\mu)\right)^2 \\ \mu &= \frac{a}{2\sqrt{\sigma^2 + D\tau}} \end{aligned} \quad (1)$$

where N is the average number of molecules in the observation area, a is the effective pixel size (320 nm with 2 × 2 pixel binning), D the diffusion coefficient, τ the lag time, and σ is the standard deviation of the microscope's point spread function (PSF) approximated as a 2D Gaussian function:

$$PSF(x,y) = exp\left(-\frac{(x^2+y^2)}{2\sigma^2}\right) \quad (2)$$

Calibration of the *PSF* was done as described in (51). We measured our *PSF* $\sigma = 0.19$ μm, corresponding to a fullwidth at half-maximum (FWHM) of 450 nm, larger than expected using a 1.46 NA oil immersion objective. This enlargement was likely caused by our use of a low-magnification tube lens that degraded resolution, as we measured a *PSF* size $\sigma = 0.16$ μm when using a higher magnification tube lens. For all acquisitions except in supported lipid bilayers (SLBs), we used an intensity threshold set to 80% of the maximum intensity in the field of view (see supporting material for the determination of intensity threshold).

Liposomes preparation

1,2-di-(9Z-Octadecenoyl)-*sn*-glycero-3-phosphocholine (DOPC) and 1-palmitoyl-2-oleoyl-*sn*-glycero-3-phosphocholine (POPC) stored in chloroform were purchased from Merck (Darmstadt, Germany) and stored under argon. Fifty microliters of 10 mg/mL stock were added to a glass tube then dried using argon under rotation. Lipids were resuspended in 1.6 mL phosphate-buffered saline (PBS), then tip-sonicated for 10 min in 30 s on/off cycles on ice. Liposomes were labeled before experiments with 1% of 1,2-dioleoyl-*sn*-glycero-3-phosphoethanolamine-*N*-(lissamine rhodamine B sulfonyl) (PE-Rhod) (Merck) at a concentration of 10 μg/mL.

SLB preparation

SLBs were prepared by liposome deposition. We pipetted 20 μL of liposome solution to a home-made microfluidic chamber made of a sandwich of a slide and a plasma-cleaned coverslip held together by two strips of molten parafilm. Excess liposomes were abundantly washed using 200 μL PBS. The chamber was then sealed using parafilm to prevent evaporation.

Beads-supported lipid bilayer preparation

Beads-supported lipid bilayers (BSLBs) were prepared as described elsewhere (52). Ten microliters of 5 μm uncoated silica beads (BioValley, Nanterre, France) were washed twice in 1 mL PBS, then mixed with 50 μL liposomes. The mix was shaken for 20 min to form BSLBs, then washed twice in 1 mL PBS. PBS (200 μL) was left after final wash, and 100 μL of BSLBs was then pipetted to a glass-bottom Ibidi chamber for imaging.

Cells preparation and staining

B. subtilis

The wild-type laboratory strain 168 *trpC2* of *B. subtilis* was grown and imaged in rich lysogeny broth medium (LB). For measurements at 37°C, 3 μL of cells from an overnight culture grown at 30°C were diluted in 2 mL LB and grown at 37°C under agitation for 2.5 h until they reached exponential phase (OD_{600} ~ 0.3) and then labeled with 0.2% (v/v) of either 50 μg/mL Nile red or 40 μg/mL Di4-ANEPPS (Thermo Fisher, Waltham, MA, USA) dissolved in DMSO (see Fig. S1 for a discussion on labeling concentration). Labeled cultures were left under agitation for an additional 15–20 min, then 3 μL was transferred to an agarose pad (1.2% in LB) for immobilization and covered with a plasma-cleaned coverslip.

For steady-state measurements at 20°C, cells from an overnight culture at 30°C were first diluted (3 μL of overnight culture diluted in 2 mL fresh LB) and grown for about 2 h at 37°C until reaching exponential phase (OD_{600} ~ 0.2), transferred at 20°C under agitation for 4–5 h for at least one generation, then labeled as described above.

S. aureus

The attenuated wild-type laboratory strain of *S. aureus* RN-4220 (53) was prepared similarly to *B. subtilis*, except for the following points. At 37°C, cells were grown until reaching exponential phase (OD_{600} ~ 0.3–0.4). Cells were labeled with 0.2% of a stock of 20 μg/mL of Nile red, lower than in *B. subtilis* as we observed phototoxicity when performing FCS experiments in *S. aureus* with a stock concentration of 50 μg/mL. For steady-state measurements at 20°C, cells were grown at 37°C to an OD_{600} of ~0.2, transferred at 20°C for 3 h for at least one generation.

Delay before acquisition

When *B. subtilis* cells were transferred from the liquid culture to the agarose-coated slide at the same temperature, we observed an initial decrease in membrane fluidity followed by membrane fluidity recovery within about 25 min (Fig. S2). This adaptation to the transfer on agarose pad could be due to osmotic shock (54), oxidative stress, or another cause that remains unknown. We therefore leave cells (both *B. subtilis* and *S. aureus*) to settle on the agarose-coated slide for 25 min before starting to image. In the case of cold shock, cells were first immobilized on an agarose pad and covered with the coverslip at 37°C for 25 min then transferred at 20°C in the microscope.

RESULTS

Filtering curves based on goodness of fit

FCS measurements can be subject to artifacts that distort FCS curves, for instance, when bright clusters of fluorescent molecules enter the observation area. These curves need to be discarded from the analysis to avoid biasing the estimation of the diffusion coefficient. In point FCS, this is often done via manual inspection facilitated by dedicated software (55). This approach is, however, impractical in imaging FCS due to the high parallelization of FCS curve acquisition resulting in the generation of a high number of FCS curves (typically we acquire 350–500 FCS curves per hour in bacteria, considering only one pixel binning value). The issue of sample-induced artifacts in FCS is well acknowledged and solutions to this issue were developed previously. It was notably proposed to compare each curve

within a data set to the averaged curve and to exclude outliers (56). This solution is, however, computationally intensive and might fail if signal levels vary within a data set, for instance, due of cell-to-cell heterogeneity. Another approach consists in rejecting curves with irregular residuals. One method for doing this consists in calculating the χ^2 goodness of fit (32), but it does not handle noisy curves well (56). It also requires knowledge of the standard deviation of the FCS curve, which is not always available. The Fourier transform was previously used to detect unevenly distributed residuals, relying, however, on fine-tuning three empirical parameters and making assumptions on the transit times observed (35). As an alternative to these methods, we introduce here a simple error metric based on fitting residuals that quantifies fitting bias. Considering the mean square error (*MSE*):

$$MSE = \frac{1}{n}\sum_{i}^{n}(r_i)^2$$
$$r_i = (y_i - \hat{y}_i)/\hat{y}_0 \tag{3}$$

where y_i is the empirical FCS curve at lag time *I*, $\hat{y}_i$ is the corresponding fit value and r_i the residual. A high *MSE* value is indicative of either a high fitting bias in a poorly fitted curve, which needs to be discarded, or of low signal value (57) caused by strong oscillations of the FCS curve around its fit. Expecting strong variations in signal levels within and across acquisitions, due to cell-to-cell variations and inhomogeneous illumination of curved cells in the TIRF field, we designed a metric that measures fit quality with a lower dependency to signal level. This metric, named nonlinear mean-square error (MSE_{nl}), is a weighted sum of residuals, where the weight of each residual is equal to the number of adjacent residuals of same sign $n_{adj,i}$:

$$MSE_{nl} = \frac{1}{n}\sum_{i}^{n} n_{adj,i}(r_i)^2 \tag{4}$$

Concretely, if residuals r_i between *i–2* and *i+2* are all positive, the fitting bias is strong and the weight $n_{adj,i} = 4$ is high. On the other hand, if the residual r_i is positive but the residuals *i–1* and *i+1* are negative, there is no fitting bias (residuals are oscillating around the mean) and $n_{adj,i} = 0$. To evaluate the capability of MSE_{nl} to evaluate fitting bias, we performed two different TIR-FCS acquisitions on a flat sample of DOPC SLBs, at either high (740 μW) or low (185 μW) excitation power. The resulting data set had heterogeneous signal levels representing the expected heterogeneity in biological samples. However, since these two acquisitions were performed on the same SLB, we expected to find a comparable number of artifactual curves with the two excitation intensities. By comparing *MSE* and MSE_{nl} for every FCS curve in the data set (Fig. 1 *A*), we could observe that excitation intensity was a good predictor of *MSE* but not of MSE_{nl}. Curves acquired at higher excitation intensity had on

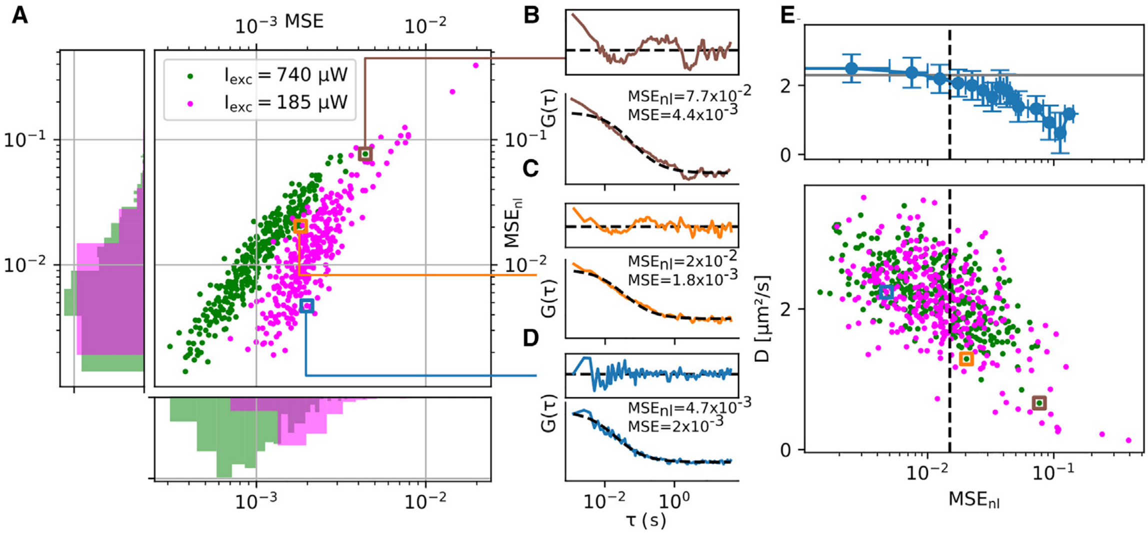

FIGURE 1 Estimation of the fit quality of FCS curves acquired on a DOPC SLB labeled with PE-Rhod using *MSE* and MSE_{nl}. (*A*) Scatterplot (*center*) of *MSE* and MSE_{nl} metrics for each curve acquired in TIR-FCS at low (*magenta*) and high (*green*) excitation powers as indicated in the legend, and histograms of the corresponding *MSE* (*bottom*) and MSE_{nl} (*left*) distributions. Colored squares refer to FCS curves shown in (*B*)–(*D*). (*B–D*) Normalized FCS curves (*color*) and fits (*dotted black lines*) with (*top*) fitting residuals, all plotted with the same scale. (*C* and *D*) FCS curves with similar *MSE* and different MSE_{nl}. (*B*) FCS curve with both higher *MSE* and MSE_{nl} than curves in (*C*) and (*D*). (*E*) Bottom: scatterplot of measured diffusion coefficient with MSE_{nl} and empirical threshold on fitting quality (*dotted vertical line*) to discard artifactual FCS curves. Squares represent FCS curves shown in (*B*)–(*D*). Top: mean ± SD of diffusion coefficient within the MSE_{nl} range for both high and low excitation intensities, represented by lateral error bars (*blue*) and (*gray*) value reported for the diffusion coefficient of PE-Rhod in a DOPC SLB in (58). To see this figure in color, go online.

average a lower *MSE* but similar MSE_{nl}, showing a lower dependence of MSE_{nl} with signal levels. We observed a strong positive correlation between the two metrics for curves acquired at a same excitation intensity (Fig. 1 *A*). This is due to poorly fitted curves having higher residuals than well-fitted curves at similar signal levels (Fig. 1, *B* and *D*). However, comparing two FCS curves with similar *MSE* but different MSE_{nl} suggested that MSE_{nl} measures fitting bias irrespective of signal levels (Fig. 1, *C* and *D*), unlike *MSE* which is heavily affected by signal levels. Considering that every FCS curve contains imperfections, we then sought to determine the maximum acceptable amount of fitting bias measured by MSE_{nl}. For this, we plotted the measured diffusion coefficient against MSE_{nl} (Fig. 1 *E*, *bottom*). We observed that high fitting biases were correlated to slower diffusion coefficients caused by artifacts in FCS curves, which could originate from temporary loss of focus or fluorescent clusters moving through the measurement area. We confirmed this by plotting the average diffusion coefficient for sets of FCS curves with MSE_{nl} thresholds (Fig. 1 *E*, *top*) and found that the average diffusion coefficient obtained with FCS curves having a low MSE_{nl} matched the diffusion coefficient previously measured on PE-Rhod in a POPC SLB (58). Using both inspection of individual FCS curves and the scatterplot shown in Fig. 1 *E*, we set the MSE_{nl} threshold to the value of 0.015. We kept this threshold throughout this study, in both synthetic and biological samples.

Impact of membrane curvature

Three assumptions made when fitting FCS curves with the model in Eq. 1 were not verified when doing TIR-FCS in bacteria. First of all, Eq. 1 assumes that the diffusion within the observation area is occurring on a 2D flat surface. TIR-FCS measures an average transit time in the observation area and then calculates a diffusion coefficient as a ratio between the size of the observation area and the transit time. In Eq. 1, it is assumed that the size of the observation area is identical to the size of the area in which molecules diffused (the diffusion area). This is true when imaging a flat surface but is not when imaging a curved surface. In the latter case, the diffusion area is larger than the observation area. Second, it is assumed that intensity fluctuations are only caused by molecules moving across the observation area and Poisson noise. However, when doing TIR-FCS in a curved membrane, molecules moving laterally also change their axial position, which, under TIRF excitation, determines excitation intensity and therefore induces intensity fluctuations. The third assumption is that the system is open, which means that there is an infinite pool of fluorescent molecules diffusing in an infinite-sized reservoir. This latest assumption is never actually verified but it is a good approximation when the mean-square displacement of fluorescent emitters during the time of acquisition is much smaller than the reservoir size. This is not the case for membrane markers in bacteria: the average distance traveled by molecules diffusing at a reasonable 1 $\mu m^2/s$ speed over the course of 1 min (our usual acquisition time) is 15 μm, larger than the characteristic dimensions of a *B. subtilis* cell (typically ~5 μm long and ~1 μm wide for exponentially growing *B. subtilis* cells in LB medium at 37°C, see Fig. S3). To evaluate potential biases in diffusion coefficient measurements caused by these effects, we simulated diffusion on curved surfaces of finite areas: either on the simplest case of a sphere (that represents *cocci*, e.g., *S. aureus*) or on a cylindrical vessel (like the rod-shaped *B. subtilis*). Diffusion on these 3D surfaces were simulated as a Wiener process. We first generated a uniform distribution of initial positions. Position vectors were updated for each step by adding the cross-product of the position vector with a random 3D vector of Brownian motion, then normalized (see supporting material for a complete description of the simulation). From the position of individual emitters determined as trajectories (Fig. 2 *A*), we could simulate TIR-FCS experiments having the physical parameters of our setup: framerate (1 ms/frame), PSF size (FWHM of 450 nm), TIRF penetration depth (100 nm). Molecular brightness was set to 20 kHz and simulated diffusion coefficient was set to 1 $\mu m^2/s$. The number of molecules was set to reach a density of 0.4 molecules/μm^2, with a minimum of 10 molecules per simulation.

Within this simulation framework, we simulated a series of TIR-FCS experiments either on rods (of constant length set to 3 μm) or on spheres of various radii (0.5–10 μm) and measured the apparent diffusion coefficient (Fig. 2 *B*). We found that, in both geometries, the measured diffusion coefficient converged toward the real value for high radius values, which was expected given that a curved membrane of high radius of curvature can be approximated as flat. The asymptotic value was nonetheless a few percent higher than the target value, which could be due to the limited number of frames or limited surface area simulated. However, we found a significant bias in diffusion coefficient measurement for small radii (below 2 μm). The measurement bias in a rod was well approximated by the square-root of the measurement bias in a sphere (Fig. 2 *B*, *dash-dotted line*). This can be explained by thinking of curvature as modifying the detection PSF: the detection PSF is modified alongside two dimensions in the case of a sphere and only one dimension in the case of a rod. FCS curves acquired in a rod shape can therefore be fitted with an updated model based on Eq. 1, which is the product of two 1D fitting models with a fitting bias *f* accounting for curvature alongside one dimension:

$$g_{xy}, rod(\tau) = \frac{1}{a^2}\left(\frac{1}{\sqrt{\pi}\mu_1}(exp(-{\mu_1}^2) - 1) + erf(\mu_1)\right)\left(\frac{1}{\sqrt{\pi}\mu_2}(exp(-{\mu_2}^2) - 1) + erf(\mu_2)\right)$$
$$\mu_1 = \frac{a}{2\sqrt{\sigma^2 + D\tau}}; \mu_2 = \frac{a}{2\sqrt{\sigma^2 + Df\tau}} \quad (5)$$

We set the fitting bias f to the value of 2.1 for *B. subtilis*, corresponding to the bias induced by a curvature of 500 nm radius on a sphere (Fig. 2 *B*). We investigated using simulations whether rod length influenced the measurement of diffusion coefficient (Fig. S10), and found that for a radius of 500 nm only rod lengths below 2.5 μm affected the diffusion measurements, which is below the lengths of *B. subtilis* cells we measured in this study (Fig. S3). To validate our simulations experimentally, we used POPC BSLBs of 5 μm diameter, labeled with the fluorescent lipid PE-Rhod, as a system of 2D diffusion of a controlled diameter (Fig. 2 *C*). We performed a series of TIR-FCS experiments in these BSLBs and measured the diffusion coefficient with different observation sizes using different pixel binning values (Fig. 2 *D*). The apparent curvature increased as the pixel binning increased and this experiment was therefore a good proxy to measure the effect of different curvatures on measured diffusion coefficient. As expected, we observed an increase in diffusion coefficient with increased observation size, corresponding to an increased effect of curvature on the effective PSF. We observed a similar change in diffusion coefficient with observation size on simulated spheres of identical diameter (Fig. 2 *E*). This analysis assumed free diffusion occurring in BSLBs, which was previously shown to not be strictly true (52). Nanoscale hindrances were previously detected in BSLBs using super-resolution spectroscopy (52), but these were unlikely to affect diffusion measurements at the larger observation scales used here. Indeed, we observed that the measured diffusion coefficient was constant for pixel sizes between 160 and 480 nm (Fig. 2 *E*), consistent with a free diffusion approximation.

Temperature-induced changes in membrane fluidity in *B. subtilis*

The fluidity of biological membranes heavily depends on the ambient temperature. Reducing temperature increases lipid order, thereby decreasing membrane fluidity, up to the point of phase transition from a fluid membrane to a gel-like structure (59). Poikilothermic ("cold-blooded") organisms like bacteria that are naturally exposed to ample changes in temperature adapt their membrane composition

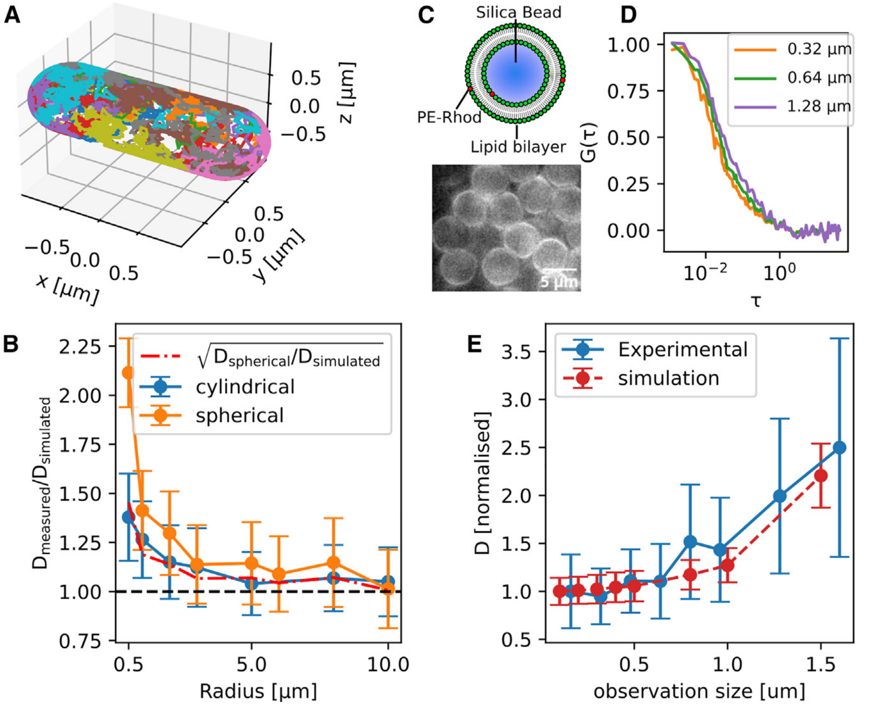

FIGURE 2 Impact of membrane curvature on TIR-FCS measurements. (*A*) 3D visualization of random trajectories generated on a rod. (*B*) Diffusion coefficients (mean ± SD) measured $D_{measured}$ from simulation of FCS measurements, normalized with simulated value $D_{simulated}$, in cylindrical (*blue*) and spherical (*orange*) coordinates. Dash-dotted red line, square root of normalized diffusion coefficient in a sphere; dotted black line, simulated diffusion coefficient (ground truth). (*C*) Cartoon of a BSLB (*top*) and epifluorescence images of BSLBs in an Ibidi chamber (*bottom*). (*D*) FCS curves for different observation sizes (obtained by pixel binning) as indicated in the legend, averaged within a 1.28 × 1.28 μm observation area. (*E*) Diffusion coefficients (mean ± SD) measured at different observation sizes, normalized with the value measured for smallest observation size from experimental measurements (*plain blue line*) in 5 μm diameter BSLBs or from simulations in a sphere of same size (*dotted red line*). To see this figure in color, go online.

to maintain fluidity to survive such changes. Bacterial membrane adaptation to low temperature has been widely studied. In the Gram-positive model organism *B. subtilis*, it primarily involves increasing the ratio of unsaturated (containing double bonds) and branched fatty acids (47,60) that have a higher melting temperature than their saturated and straight-chain counterparts, resulting in more fluid membranes. The plasma membrane of exponentially growing *B. subtilis* cells in rich medium contains only low amounts of unsaturated fatty acids (47,61). Upon cold shock, the two-component system DesK/DesR is activated by an increase of membrane thickness (62), which in turn triggers the expression of the *des* gene (6), coding for the fatty acid desaturase Des that desaturates fatty acids (60) immediately (<30 min) (46). Longer-term membrane re-adaptation involves branching instead of saturation and a decrease in average fatty acid chain length (47). The resulting changes in membrane fluidity can, however, not directly be calculated from the fatty acid chain composition alone, as other changes come into play and the membrane fluidification by fatty acid saturation and branching at low temperature is compensated to an unknown extent by a loss of fluidity caused by a temperature decrease.

We thus aimed to measure membrane fluidity of *B. subtilis* cells growing at either 37 or 20°C using our TIR-FCS assay. We used the membrane dye Nile red, which is widely used in *B. subtilis* and has the advantage of being bright and photostable. Cells growing in liquid were stained, immobilized on an agarose-coated slide and allowed to stabilize for 25 min before imaging (see Fig. S2 and supporting material for details). Individual diffusion maps alone clearly showed that the diffusion speed of Nile red was slower at 20 than at 37°C (Fig. 3, *A* and *B*). This observation was verified through multiple acquisitions, showing an about twofold reduction in the diffusion coefficient of Nile red at 20°C relative to 37°C (Fig. 3 *C*). We verified that cells were healthy and exponentially growing by monitoring their growth before imaging (Fig. S4). The rare cells that were not growing in the microscopy field were excluded from the analysis. We also estimated the impact of phototoxicity by monitoring the growth at the single-cell level after imaging and found that cells were growing after imaging, although at a slightly slower rate (Fig. S4). To verify whether the diffusion speed of Nile red was controlled by membrane fluidity and not by unforeseen interactions, we performed the same experiment with the membrane dye Di4-ANEPPS (Di4). Diffusion speed of Di4 was slower than Nile red in similar experimental conditions (Fig. 3 *C*). This could be caused by its larger size (M = 318 g/mol for Nile red and 480 g/mol for Di4), different location in the membrane, or another unknown factor. The reduction of diffusion speed measured between 37 and 20°C was

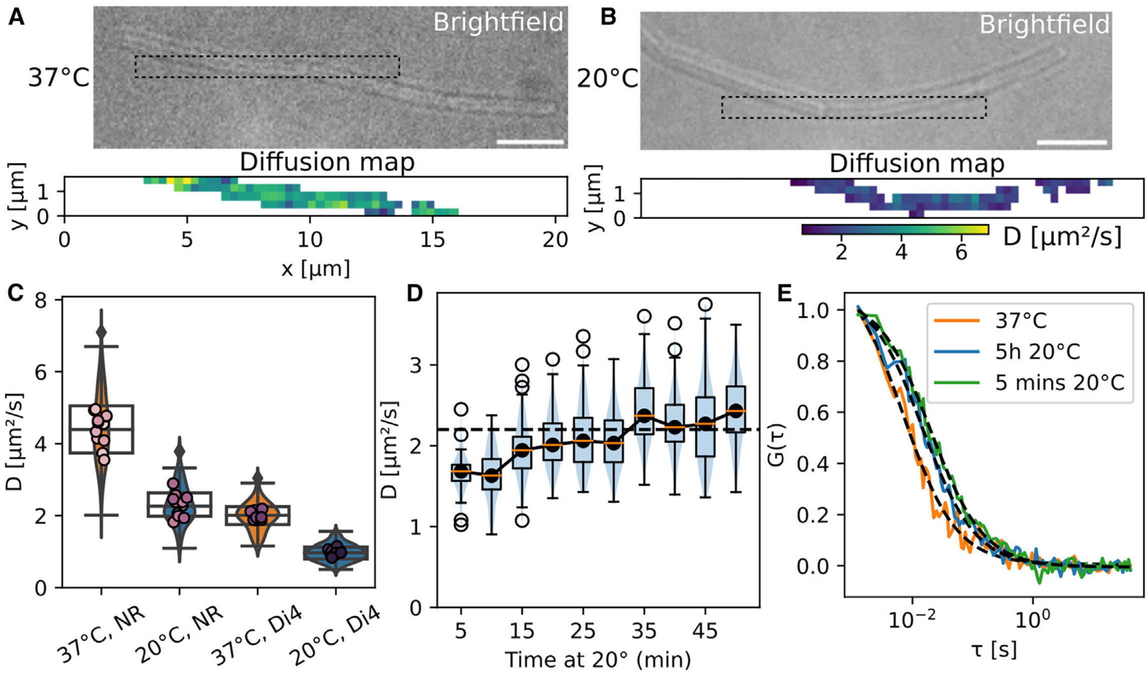

FIGURE 3 Adaptation of *B. subtilis* membrane to cold temperature, measured with TIR-FCS. (*A* and *B*) representative brightfield (*top*) with highlighted areas (*dotted squares*) where TIR-FCS diffusion maps (*bottom*) of Nile red in exponentially growing *B. subtilis* were acquired, at 37°C (*A*) or after 5 h at 20°C (*B*). Scale bars, 5 μm. (*C*) Diffusion coefficient of Nile red (*left*) and Di4 (*right*) in *B. subtilis* cells grown and imaged at 37°C (*orange*) or 5 h after transfer at 20°C (*blue*). Dots: average of individual TIR-FCS acquisitions of one or more cells, two biological replicates, $n > 280$ FCS curves per condition. (*D*) Recovery of diffusion speed of Nile red upon transfer from 37 to 20°C, pooling measurements by tranches of 5 min, $n = 2$ biological replicates. Dotted black line: median diffusion coefficient of Nile red at 20°C from (*C*). (*E*) Normalized FCS curves obtained at 37°C (*orange*) and 5 min (*green*) and 5 h (*blue*) after transfer at 20°C. To see this figure in color, go online.

identical for both dyes (Fig. 3 *C*; Table 1), suggesting that it was indeed a change in fluidity that led to the observed reductions in diffusion coefficient.

Having established a baseline for the diffusivity of Nile red in *B. subtilis* at different temperatures, we then sought to monitor the remodeling of the membrane in response to a cold shock. Cells growing exponentially at 37°C were labeled with Nile red, immobilized on an agarose pad, and then transferred to a thermostated microscope chamber at 20°C. We then performed TIR-FCS acquisitions on different cells for 1 h. As expected from a sudden temperature downshift, we observed first a reduction in diffusion coefficient (Fig. 3, *D* and *E*). Then, the diffusion coefficient progressively increased, likely caused by membrane adaptation, until reaching after ~25–30 min the steady-state value previously measured for cells growing at 20°C (Fig. 3 *C*). This timescale of membrane fluidity adaptation is consistent with the significant membrane fatty acids remodeling within 30 min after a cold shock that was reported previously (46). The diffusion coefficients measured were fitted using the model of Eq. 5 accounting for membrane curvature, assuming a constant radius of 0.5 μm and a cell length larger than 2.5 μm (see Fig. S10). Manual measurements of cell width and length confirmed these hypotheses (Fig. S3).

Temperature-induced changes in membrane fluidity in *S. aureus*

We then set out to measure the membrane fluidity during cold shock of a different bacterium with a different geometry, the spherical Gram-positive pathogen *S. aureus*. Cold shock response has been thoroughly characterized in *B. subtilis* but is poorly understood in *S. aureus*. It was mainly found that, upon a drop in temperature, the membrane of *S. aureus* becomes enriched in carotenoids (63) and that cold shock stabilizes most of its RNA species (64). More recently, an analog of the *B. subtilis* two-component system DesK/DesR and transcription of *des* (encoding the fatty acid desaturase Des) responsible for temperature sensing was found in *S. aureus* (65), suggesting a similar response to cold shock in both bacteria. To confirm this, we conducted in *S. aureus* a series of experiments similar to the ones we performed in *B. subtilis*. Using TIR-FCS, we measured the diffusion speed of Nile red in the membrane of growing *S. aureus* cells at 37 and 20°C (Fig. 4 *A*). FCS curves were fitted with the standard TIR-FCS

TABLE 1 Diffusion coefficients of Nile red and Di4-ANEPPS measured in *B. subtilis* cells growing at 37 and 20°C, and ratios of diffusion coefficients at 37 and 20°C

Dye	$D_{37^\circ C}$ (mean ± SD)	$D_{20^\circ C}$ (mean ± SD)	$D_{37^\circ C}/D_{20^\circ C}$
Nile red	4.4 ± 0.3	2.2 ± 0.2	2 ± 0.3
Di4-ANEPPS	1.9 ± 0.1	0.9 ± 0.07	2.1 ± 0.3

Presented are the means and standard deviations of the average diffusion coefficients for each acquisition (*dots* in Fig. 3 *C*).

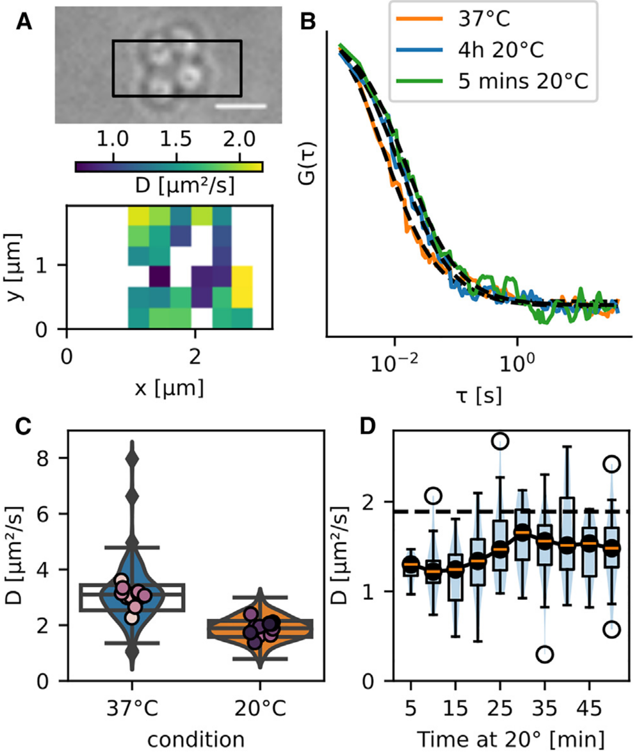

FIGURE 4 Adaptation of *S. aureus* membrane to cold temperature, measured with TIR-FCS. (*A*) Diffusion map of Nile red in *S. aureus* (*bottom*) and corresponding bright-field image (*top*, measured area in *black square*, scale bar, 2 μm) at 20°C. (*B*) Normalized FCS curves obtained at 37°C (*orange*) and 5 min (*green*) and 4 h (*blue*) after transfer at 20°C. (*C*) Diffusion coefficient of Nile red in *S. aureus* cells grown and imaged at 37°C (*orange*) or 4 h after transfer at 20°C (*blue*). Dots: average of individual TIR-FCS acquisitions of one or more cells, two to three biological replicates, $n > 150$ FCS curves per condition. (*D*) Recovery of diffusion speed of Nile red upon transfer from 37 to 20°C, pooling measurements by tranches of 5 min, $n = 3$ biological replicates. Dotted black line: median diffusion coefficient of Nile red at 20°C from (*C*). To see this figure in color, go online.

model (Eq. 1) and corrected for curvature using the simulated measurement bias calculated in Fig. 2 *B*. In practice, diffusion coefficients were divided by a factor of 2.1, corresponding to the bias induced by a sphere of radius 500 nm, corresponding to the radius of *S. aureus* we observed in our experiments (Fig. S5). For all conditions, we confirmed that cells kept growing on the slide before imaging, and we excluded cells that were not growing from the analysis. As expected, diffusion speed in steady-state measurements was higher at 37°C than at 20°C, like in *B. subtilis*, with, however, a slightly different ratio (Fig. 4 *C*; Table 2). We measured a similar diffusion coefficient of Nile red at 20°C for *B. subtilis* and *S. aureus* but a higher diffusion

TABLE 2 Diffusion coefficients of Nile red in *S. aureus* at 37 and 20°C, and ratio of both diffusion coefficients

$D_{37^\circ C}$ (mean ± SD)	$D_{20^\circ C}$ (mean ± SD)	$D_{37^\circ C}/D_{20^\circ C}$
3.0 ± 0.3	1.9 ± 0.3	1.7 ± 0.4

coefficient in *B. subtilis* at 37°C. When *S. aureus* cells were transferred from 37 to 20°C, we observed first a loss of fluidity due to the sudden change in temperature, followed by a recovery in fluidity to reach a plateau after ∼25–30 min, as also observed in *B. subtilis* (Fig. 4 *D*). The average diffusion coefficient of Nile red did not recover up to the 20°C steady-state value as in *B. subtilis* (Figs. 4 *D* and 3 *D*, respectively). We assumed that the recovery of membrane fluidity is nonetheless complete within 30 min and that the lower average diffusion coefficient we measured was caused by statistical variations between replicates (Fig. S6).

DISCUSSION

In this paper, we demonstrate how TIR-FCS can be used to quantitatively measure membrane fluidity in bacteria, exemplified by the Gram-positive model organism *B. subtilis* and *S. aureus*. For this, we derived a new fit quality metric that greatly simplified data analysis. Using simulations validated by experiments, we estimated the measurement bias caused by the observation of a curved membrane of finite size in a TIRF field, and used this information to perform unbiased measurements of diffusion coefficients in the membrane of spherical and rod-shaped bacterial cells. We used this assay to measure membrane fluidity, reported by diffusion speed of membrane markers, in the membrane of *B. subtilis* and *S. aureus* at different temperatures, in steady state, and during membrane remodeling caused by a cold shock.

The fitting quality metric that we proposed is a fine addition to the collection of readily available quality metrics for FCS quality assessment. Its reliability across data sets with different signal levels might prove useful in future high-throughput FCS studies, whether camera based or not (66). Using TIR-FCS instead of conventional confocal FCS offered several advantages, including high-throughput measurements, excellent axial selectivity, allowing FCS measurements in single membranes and extraction of spatial information. Besides, the use of an unpolarized evanescent field with TIRF excitation allows excitation of fluorescent molecules irrespective of their dipole orientation. This is a major advantage when working with membrane markers such as Nile red or Di4 that intercalate within a membrane and keep a fixed orientation, which can be orthogonal to the polarization plane of, e.g., confocal excitation, and therefore lead to poor excitation quality and inhomogeneous excitation of observation area.

Simulating TIR-FCS in curved surfaces proved very useful to understand and measure the potential biases induced by bacterial membrane curvature, inhomogeneous excitation of the curved membrane in the evanescent field, and diffusion in a closed system of small size. The respective effect of each one these three parameters was not disentangled by our simulations. Further investigations would be required to quantify their individual contributions to the final measurement. Simple 2D simulations in finite-sized boxes could readily reveal that the small diffusion areas typical of bacterial membranes can lead to substantial biases in measured diffusion speed (Fig. S9). Care must be taken when accounting for membrane curvature with simulations to accurately simulate not only the observed geometry, but also the microscope itself as parameters such as the PSF size affect the final bias in diffusion coefficient (Fig. S10). Furthermore, we did not account for photobleaching in our simulations. This might affect the measurement bias caused by the closedness of the system: in a closed system, the same molecule is more likely to diffuse repeatedly through an observation area than in an open system. However, if there is significant bleaching, a molecule will likely diffuse only once through the observation area and then bleach. We do not expect this effect to significantly change the predictions of our simulations: bleaching was not simulated but occurred in BSLBs, and both systems exhibited a similar behavior (Fig. 2 *E*).

In this work, we have shown that in *B. subtilis* the diffusivity of two different membrane markers decreased similarly in response to a decrease in temperature, suggesting that membrane fluidity is the common determinant to their diffusivity. Claiming that our assay measures membrane fluidity requires, however, a couple of reasonable approximations. Membrane fluidity in the field of microbiology is usually referred to as a single parameter (the physical parameter governing diffusion speed in the plasma membrane). However, it is known that the fluidity in the physical sense affects the diffusivity of objects depending on their hydrophobic radius (67). The diffusivity of the membrane markers Nile red and Di4 is therefore a good proxy for the measurement of membrane fluidity experienced by molecules of comparable size (e.g., lipids). However, the fluidity experienced by larger molecules such as transmembrane or membrane-associated proteins is different. Relative differences in the diffusivity of transmembrane proteins and membrane markers between experimental conditions should nevertheless remain the same, provided that membrane fluidity is the main driver of their diffusivity. In *B. subtilis*, it was found using SPT that the diffusivity of a series of membrane proteins varied as expected with temperature-induced membrane fluidity variations and depended otherwise mostly on their number of transmembrane domains (68). However, interactions with nonmembrane cellular components may also affect the diffusivity of proteins, for instance, for proteins that are partly embedded in the cell wall through an intrinsically disordered region (69): in this case, cell wall properties are likely the main regulator of their diffusion speed. The slower diffusion speed of Nile red at 20°C relative to 37°C indicates that membrane proteins undergoing Brownian motion also diffuse more slowly at 20°C than at 37°C. This could be verified by performing a series of similar TIR-FCS experiments to measure the diffusivity of fluorescent protein fusions instead of membrane markers. Furthermore, we did not consider

lateral membrane heterogeneities (named functional microdomains in bacteria (70), conceptually equivalent to lipid rafts (71) in eukaryotes) that are thought to recruit certain proteins and lipids in areas of higher molecular order and therefore lower mobility. The diffusivity that we measured in this study likely represents an average of the diffusivity in all domains of the membrane.

Our measurements were performed in Gram-positive bacteria, which lack an outer membrane. More care will need to be taken to perform TIR-FCS experiments on membrane markers in Gram-negative bacteria. First, the fluorescent marker used should label only the membrane of interest, either the inner or the outer membrane. Second, TIR-FCS experiments will be precluded in experimental conditions where the topology of the membrane is affected, such as in presence of certain antibiotics (28,72), as changes in membrane topology significantly bias diffusion coefficient estimation with FCS (73).

Our *B. subtilis* TIR-FCS data confirm the effect of a cold shock on membrane fluidity previously reported using fatty acid analysis and environment-sensitive dyes (47,74). Upon cold shock, membrane fluidity initially decreases but rapidly increases again due to a modification of plasma membrane composition that involves an increase in unsaturated and branched-chain fatty acids in the membrane (47). However, it remained unknown to what extent membrane fluidity recovered. Fatty acid profiles only provided qualitative information and conflicting results were obtained with environment-sensitive probes (74). DPH anisotropy suggested an incomplete recovery of *B. subtilis* membrane fluidity while fluorescence lifetime measurements of the same probe indicated an identical fluidity at 20 and 37°C (74). Our results allow to settle this debate unambiguously: after a cold shock, membrane fluidity does not completely recover to the precold shock value in *B. subtilis*, it recovers to the steady-state fluidity at the temperature to which cells were transferred.

We have demonstrated the ability of our assay to measure membrane fluidity in coccoid bacteria as well by studying the diffusivity of Nile red in the membrane of *S. aureus*. Little was known about the adaptation of the plasma membrane of *S. aureus* to a cold shock, except that the membrane gets enriched in carotenoids (63) and that a thermosensor resembling that of *B. subtilis* (the DesK/DesR two-component system) is expected to regulate plasma membrane fluidity (65). Our work revealed a similar recovery time after cold shock in both bacteria, both occurring within ~30 min. As in *B. subtilis*, the fluidity of *S. aureus* recovers to its steady-state value at 20°C, which is lower than its value at 37°C. Our measurements also revealed that membrane fluidity is lower in *S. aureus* than in *B. subtilis* at 37°C and similar in both species at 20°C. Therefore, the quantitative and purely physical measure provided by our assay also allows for interspecies comparison.

Why do *B. subtilis* and *S. aureus* not maintain the same membrane fluidity across temperatures? An explanation might be that membrane fluidity homeostasis occurs only below a certain critical temperature, below which the plasma membrane transitions to a deadly gel-like phase. This is supported by previous findings that activation of the DesK thermosensor in *B. subtilis* occurs only below ~30°C (75,76). In this scenario, membrane fluidity would be regulated only at low temperatures to prevent fluidity from dropping below a certain critical threshold. Temperature increases above 30°C would not lead to changes in membrane composition, leading to an increase in membrane fluidity caused by thermodynamics.

In conclusion, our TIR-FCS assay opens up exciting perspectives in the field of microbiology. The unprecedented ability to directly quantify membrane fluidity will shed a new light on the biophysics of bacterial membranes and might help to understand key cellular processes such as membrane remodeling upon viral infection or changes in nutriment availability, as well as the mode of action of membrane-targeting antibiotics. This newly acquired capability to quantify membrane fluidity will also enhance our understanding of the fundamental role of membrane fluidity in bacterial physiology.

DATA AND CODE AVAILABILITY

Research data are available on Zenodo: https://doi.org/10.5281/zenodo.11236214. Code and manual of the FCS analysis software developed for this project and used throughout this paper can be found at https://github.com/aurelien-barbotin/pyimfcs. Code for simulations can be found at https://github.com/aurelien-barbotin/geomdsim.

SUPPORTING MATERIAL

Supporting material can be found online at https://doi.org/10.1016/j.bpj.2024.06.012.

AUTHOR CONTRIBUTIONS

A.B., C.B., E.S., and R.C.-L. designed the project and wrote the manuscript. A.B. performed the experiment, analyzed data, and wrote the code.

ACKNOWLEDGMENTS

This project has received funding from the European Research Council (ERC) under the Horizon 2020 Research and Innovation Program (grant agreement no. 772178 to R.C.-L.) and under the Marie Sklodowska-Curie grant agreement no. 101030628. E.S. is supported by Swedish Research Council Starting Grant (2020-02682). The authors thank Dr Alexandra Gruss who provided us with the *S. aureus* strain RN-4220-R. We also thank the SciLifeLab Advanced Light Microscopy Facility and National Microscopy Infrastructure (VR-RFI 2016-00968) for their support on imaging.

DECLARATION OF INTERESTS

The authors declare no competing interests.

REFERENCES

1. Blouin, C. M., Y. Hamon, …, C. Lamaze. 2016. Glycosylation-Dependent IFN-γR Partitioning in Lipid and Actin Nanodomains Is Critical for JAK Activation. *Cell.* 166:920–934.
2. Sezgin, E., Y. Azbazdar, …, G. Ozhan. 2017. Binding of canonical Wnt ligands to their receptor complexes occurs in ordered plasma membrane environments. *FEBS J.* 284:2513–2526.
3. Makarova, M., M. Peter, …, S. Oliferenko. 2020. Delineating the Rules for Structural Adaptation of Membrane-Associated Proteins to Evolutionary Changes in Membrane Lipidome. *Curr. Biol.* 30:367–380.e8.
4. Dewald, A. H., J. C. Hodges, and L. Columbus. 2011. Physical Determinants of β-Barrel Membrane Protein Folding in Lipid Vesicles. *Biophys. J.* 100:2131–2140.
5. Burgess, N. K., T. P. Dao, …, K. G. Fleming. 2008. Beta-barrel proteins that reside in the Escherichia coli outer membrane in vivo demonstrate varied folding behavior in vitro. *J. Biol. Chem.* 283:26748–26758.
6. Vaňousová, K., J. Beranová, …, I. Konopásek. 2018. Membrane fluidization by alcohols inhibits DesK-DesR signalling in Bacillus subtilis. *Biochim. Biophys. Acta Biomembr.* 1860:718–727.
7. Popp, P. F., A. Benjdia, …, T. Mascher. 2020. The Epipeptide YydF Intrinsically Triggers the Cell Envelope Stress Response of Bacillus subtilis and Causes Severe Membrane Perturbations. *Front. Microbiol.* 11:151.
8. Lee, T.-H., V. Hofferek, …, M.-I. Aguilar. 2019. The role of bacterial lipid diversity and membrane properties in modulating antimicrobial peptide activity and drug resistance. *Curr. Opin. Chem. Biol.* 52:85–92.
9. Loffeld, B., and H. Keweloh. 1996. cis/trans isomerization of unsaturated fatty acids as possible control mechanism of membrane fluidity in Pseudomonas putida P8. *Lipids.* 31:811–815.
10. Beney, L., and P. Gervais. 2001. Influence of the fluidity of the membrane on the response of microorganisms to environmental stresses. *Appl. Microbiol. Biotechnol.* 57:34–42.
11. Budin, I., T. De Rond, …, J. D. Keasling. 2018. Viscous control of cellular respiration by membrane lipid composition. *Science.* 362:1186–1189.
12. Sáenz, J. P., D. Grosser, …, K. Simons. 2015. Hopanoids as functional analogues of cholesterol in bacterial membranes. *Proc. Natl. Acad. Sci. USA.* 112:11971–11976.
13. Boudjemaa, R., C. Cabriel, …, K. Steenkeste. 2018. Impact of Bacterial Membrane Fatty Acid Composition on the Failure of Daptomycin To Kill *Staphylococcus aureus*. *Antimicrob. Agents Chemother.* 62:e00233-18.
14. Nielsen, L. E., D. R. Kadavy, …, K. W. Nickerson. 2005. Survey of Extreme Solvent Tolerance in Gram-Positive Cocci: Membrane Fatty Acid Changes in *Staphylococcus haemolyticus* Grown in Toluene. *Appl. Environ. Microbiol.* 71:5171–5176.
15. Liu, S., S. Brul, and S. A. J. Zaat. 2021. Isolation of Persister Cells of Bacillus subtilis and Determination of Their Susceptibility to Antimicrobial Peptides. *Int. J. Mol. Sci.* 22:10059.
16. Willdigg, J. R., and J. D. Helmann. 2021. Mini Review: Bacterial Membrane Composition and Its Modulation in Response to Stress. *Front. Mol. Biosci.* 8:634438.
17. Yoon, Y., H. Lee, …, K.-H. Choi. 2015. Membrane fluidity-related adaptive response mechanisms of foodborne bacterial pathogens under environmental stresses. *Food Res. Int.* 72:25–36.
18. Mendoza, D. D. 2014. Temperature Sensing by Membranes. *Annu. Rev. Microbiol.* 68:101–116.
19. Zielińska, A., A. Savietto, …, D.-J. Scheffers. 2020. Flotillin-mediated membrane fluidity controls peptidoglycan synthesis and MreB movement. *Elife.* 9:e57179.
20. Nickels, J. D., S. Poudel, …, J. G. Elkins. 2020. Impact of Fatty-Acid Labeling of Bacillus subtilis Membranes on the Cellular Lipidome and Proteome. *Front. Microbiol.* 11:914.
21. Strahl, H., F. Bürmann, and L. W. Hamoen. 2014. The actin homologue MreB organizes the bacterial cell membrane. *Nat. Commun.* 5:3442.
22. Nichols, C. M., J. P. Bowman, and J. Guezennec. 2005. Effects of Incubation Temperature on Growth and Production of Exopolysaccharides by an Antarctic Sea Ice Bacterium Grown in Batch Culture. *Appl. Environ. Microbiol.* 71:3519–3523.
23. Chattopadhyay, M. K. 2006. Mechanism of bacterial adaptation to low temperature. *J. Bio. Sci.* 31:157–165.
24. Edgcomb, M. R., S. Sirimanne, …, R. D. Morse. 2000. Electron paramagnetic resonance studies of the membrane fluidity of the foodborne pathogenic psychrotroph Listeria monocytogenes. *Biochim. Biophys. Acta.* 1463:31–42.
25. Konings, A. W., and A. C. Ruifrok. 1985. Role of Membrane Lipids and Membrane Fluidity in Thermosensitivity and Thermotolerance of Mammalian Cells. *Radiat. Res.* 102:86–98.
26. Saxton, M. J., and K. Jacobson. 1997. SINGLE-PARTICLE TRACKING:Applications to Membrane Dynamics. *Annu. Rev. Biophys. Biomol. Struct.* 26:373–399.
27. Devkota, R., and M. Pilon. 2018. FRAP: A Powerful Method to Evaluate Membrane Fluidity in *Caenorhabditis elegans*. *Bio. Protoc.* 8:e2913.
28. Ponmalar, I. I., J. Swain, and J. K. Basu. 2022. Modification of bacterial cell membrane dynamics and morphology upon exposure to sub inhibitory concentrations of ciprofloxacin. *Biochim. Biophys. Acta Biomembr.* 1864:183935.
29. Ragaller, F., L. Andronico, …, E. Sezgin. 2022. Dissecting the mechanisms of environment sensitivity of smart probes for quantitative assessment of membrane properties. *Open Biol.* 12:220175.
30. Amaro, M., F. Reina, …, E. Sezgin. 2017. Laurdan and Di-4-ANEPPDHQ probe different properties of the membrane. *J. Phys. D Appl. Phys.* 50:134004.
31. Poojari, C., N. Wilkosz, …, T. Róg. 2019. Behavior of the DPH fluorescence probe in membranes perturbed by drugs. *Chem. Phys. Lipids.* 223:104784.
32. Meacci, G., J. Ries, …, K. Kruse. 2006. Mobility of Min-proteins in *Escherichia coli* measured by fluorescence correlation spectroscopy. *Phys. Biol.* 3:255–263.
33. Cluzel, P., M. Surette, and S. Leibler. 2000. An Ultrasensitive Bacterial Motor Revealed by Monitoring Signaling Proteins in Single Cells. *Science.* 287:1652–1655.
34. Dajkovic, A., E. Hinde, …, R. Carballido-Lopez. 2016. Dynamic Organization of SecA and SecY Secretion Complexes in the B. subtilis Membrane. *PLoS One.* 11:e0157899.
35. Guet, C. C., L. Bruneaux, …, P. Cluzel. 2008. Minimally invasive determination of mRNA concentration in single living bacteria. *Nucleic Acids Res.* 36:e73.
36. Diepold, A., E. Sezgin, …, J. P. Armitage. 2017. A dynamic and adaptive network of cytosolic interactions governs protein export by the T3SS injectisome. *Nat. Commun.* 8:15940.
37. Barbotin, A., I. Urbančič, …, E. Sezgin. 2020. z-STED Imaging and Spectroscopy to Investigate Nanoscale Membrane Structure and Dynamics. *Biophys. J.* 118:2448–2457.
38. Kannan, B., J. Y. Har, …, T. Wohland. 2006. Electron Multiplying Charge-Coupled Device Camera Based Fluorescence Correlation Spectroscopy. *Anal. Chem.* 78:3444–3451.
39. Kannan, B., L. Guo, …, T. Wohland. 2007. Spatially Resolved Total Internal Reflection Fluorescence Correlation Microscopy Using an Electron Multiplying Charge-Coupled Device Camera. *Anal. Chem.* 79:4463–4470.
40. Bag, N., D. A. Holowka, and B. A. Baird. 2020. Imaging FCS delineates subtle heterogeneity in plasma membranes of resting mast cells. *Mol. Biol. Cell.* 31:709–723.
41. Ng, X. W., C. Teh, …, T. Wohland. 2016. The Secreted Signaling Protein Wnt3 Is Associated with Membrane Domains In Vivo: A SPIM-FCS Study. *Biophys. J.* 111:418–429.
42. Ng, J., R. D. Kamm, …, R. S. Kraut. 2018. Evidence from ITIR-FCS Diffusion Studies that the Amyloid-Beta (Aβ) Peptide Does Not

Perturb Plasma Membrane Fluidity in Neuronal Cells. *J. Mol. Biol.* 430:3439–3453.

43. Bag, N., D. H. X. Yap, and T. Wohland. 2014. Temperature dependence of diffusion in model and live cell membranes characterized by imaging fluorescence correlation spectroscopy. *Biochim. Biophys. Acta.* 1838:802–813.
44. Yao, Z., and R. Carballido-López. 2014. Fluorescence Imaging for Bacterial Cell Biology: From Localization to Dynamics, From Ensembles to Single Molecules. *Annu. Rev. Microbiol.* 68:459–476.
45. Wawrezinieck, L., H. Rigneault, …, P.-F. Lenne. 2005. Fluorescence Correlation Spectroscopy Diffusion Laws to Probe the Submicron Cell Membrane Organization. *Biophys. J.* 89:4029–4042.
46. Konopasek, I., K. Strzalka, and J. Svobodova. 2000. Cold shock in Bacillus subtilis: different effects of benzyl alcohol and ethanol on the membrane organisation and cell adaptation. *Biochim. Biophys. Acta.* 1464:18–26.
47. Beranova, J., M. C. Mansilla, …, I. Konopasek. 2010. Differences in Cold Adaptation of Bacillus subtilis under Anaerobic and Aerobic Conditions. *J. Bacteriol.* 192:4164–4171.
48. Ries, J., S. Chiantia, and P. Schwille. 2009. Accurate Determination of Membrane Dynamics with Line-Scan FCS. *Biophys. J.* 96:1999–2008.
49. Müller, P. 2012. Python Multiple-Tau Algorithm (Version 0.3.3) [Computer program]. https://pypi.python.org/pypi/multipletau/.
50. Ries, J., E. P. Petrov, and P. Schwille. 2008. Total Internal Reflection Fluorescence Correlation Spectroscopy: Effects of Lateral Diffusion and Surface-Generated Fluorescence. *Biophys. J.* 95:390–399.
51. Bag, N., J. Sankaran, …, T. Wohland. 2012. Calibration and Limits of Camera-Based Fluorescence Correlation Spectroscopy: A Supported Lipid Bilayer Study. *ChemPhysChem.* 13:2784–2794.
52. Beckers, D., D. Urbancic, and E. Sezgin. 2020. Impact of Nanoscale Hindrances on the Relationship between Lipid Packing and Diffusion in Model Membranes. *J. Phys. Chem. B.* 124:1487–1494.
53. Pathania, A., J. Anba-Mondoloni, …, A. Gruss. 2021. ppGpp/GTP and Malonyl-CoA Modulate Staphylococcus aureus Adaptation to FASII Antibiotics and Provide a Basis for Synergistic Bi-Therapy. *mBio.* 12:e03193-20.
54. Los, D. A., and N. Murata. 2004. Membrane fluidity and its roles in the perception of environmental signals. *Biochim. Biophys. Acta.* 1666:142–157.
55. Waithe, D., F. Schneider, …, C. Eggeling. 2018. Optimized processing and analysis of conventional confocal microscopy generated scanning FCS data. *Methods.* 140–141:62–73.
56. Ries, J., M. Bayer, …, P. Schwille. 2010. Automated suppression of sample-related artifacts in Fluorescence Correlation Spectroscopy. *Opt Express.* 18:11073–11082.
57. Schneider, F., P. Hernandez-Varas, …, I. Urbančič. 2020. High photon count rates improve the quality of super-resolution fluorescence fluctuation spectroscopy. *J. Phys. D Appl. Phys.* 53:164003.
58. Tang, W. H., S. R. Sim, …, T. Wohland. 2023. Deep learning reduces data requirements and allows real-time measurements in Imaging Fluorescence Correlation Spectroscopy. *Biophys. J.* 123:655–666.
59. Heimburg, T. 2019. Phase transitions in biological membranes. Preprint at arXiv. https://doi.org/10.48550/arXiv:1805.11481v1.
60. Mansilla, M. C., and D. de Mendoza. 2005. The Bacillus subtilis desaturase: a model to understand phospholipid modification and temperature sensing. *Arch. Microbiol.* 183:229–235.
61. Suutari, M., and S. Laakso. 1992. Unsaturated and branched chain-fatty acids in temperature adaptation of Bacillus subtilis and Bacillus megaterium. *Biochim. Biophys. Acta.* 1126:119–124.
62. Cybulski, L. E., J. Ballering, …, J. A. Killian. 2015. Activation of the bacterial thermosensor DesK involves a serine zipper dimerization motif that is modulated by bilayer thickness. *Proc. Natl. Acad. Sci. USA.* 112:6353–6358.
63. Joyce, G. H., R. K. Hammond, and D. C. White. 1970. Changes in Membrane Lipid Composition in Exponentially Growing Staphylococcus aureus During the Shift from 37 to 25 C. *J. Bacteriol.* 104:323–330.
64. Anderson, K. L., C. Roberts, …, P. M. Dunman. 2006. Characterization of the Staphylococcus aureus Heat Shock, Cold Shock, Stringent, and SOS Responses and Their Effects on Log-Phase mRNA Turnover. *J. Bacteriol.* 188:6739–6756.
65. Fernández, P., A. R. Díaz, …, M. C. Mansilla. 2020. Identification of Novel Thermosensors in Gram-Positive Pathogens. *Front. Mol. Biosci.* 7:592747.
66. Wachsmuth, M., C. Conrad, …, J. Ellenberg. 2015. High-throughput fluorescence correlation spectroscopy enables analysis of proteome dynamics in living cells. *Nat. Biotechnol.* 33:384–389.
67. Petrov, E. P., and P. Schwille. 2008. Translational diffusion in lipid membranes beyond the Saffman-Delbruck approximation. *Biophys. J.* 94:41–43.
68. Lucena, D., M. Mauri, …, P. L. Graumann. 2018. Microdomain formation is a general property of bacterial membrane proteins and induces heterogeneity of diffusion patterns. *BMC Biol.* 16:97.
69. Brunet, Y. R., C. Habib, …, D. Z. Rudner. 2022. Intrinsically disordered protein regions are required for cell wall homeostasis in Bacillus subtilis. *Genes Dev.* 36:970–984.
70. Bramkamp, M., and D. Lopez. 2015. Exploring the Existence of Lipid Rafts in Bacteria. *Microbiol. Mol. Biol. Rev.* 79:81–100.
71. Sezgin, E., I. Levental, …, C. Eggeling. 2017. The mystery of membrane organization: composition, regulation and roles of lipid rafts. *Nat. Rev. Mol. Cell Biol.* 18:361–374.
72. Manioglu, S., S. M. Modaresi, …, S. Hiller. 2022. Antibiotic polymyxin arranges lipopolysaccharide into crystalline structures to solidify the bacterial membrane. *Nat. Commun.* 13:6195.
73. Gesper, A., S. Wennmalm, …, I. Parmryd. 2020. Variations in Plasma Membrane Topography Can Explain Heterogenous Diffusion Coefficients Obtained by Fluorescence Correlation Spectroscopy. *Front. Cell Dev. Biol.* 8:767.
74. Heřman, P., I. Konopásek, …, J. Svobodová. 1994. Time-resolved polarized fluorescence studies of the temperature adaptation in Bacillus subtilis using DPH and TMA-DPH fluorescent probes. *Biochim. Biophys. Acta.* 1190:1–8.
75. Cybulski, L. E., M. Martín, …, D. de Mendoza. 2010. Membrane Thickness Cue for Cold Sensing in a Bacterium. *Curr. Biol.* 20:1539–1544.
76. Aguilar, P. S., A. M. Hernandez-Arriaga, …, D. de Mendoza. 2001. Molecular basis of thermosensing: a two-component signal transduction thermometer in Bacillus subtilis. *EMBO J.* 20:1681–1691.

Biophysical Journal, Volume *123*

Supplemental information

Quantification of membrane fluidity in bacteria using TIR-FCS

Aurélien Barbotin, Cyrille Billaudeau, Erdinc Sezgin, and Rut Carballido-López

Quantification of membrane fluidity in bacteria using TIR-FCS: Supporting material

Aurélien Barbotin[1, *], Cyrille Billaudeau[1] ,Erdinc Sezgin[2], Rut Carballido-López[1, *]

1 Université Paris-Saclay, INRAE, AgroParisTech, Micalis Institute, 78350, Jouy-en-Josas, France.
2 Science for Life Laboratory, Department of Women's and Children's Health, Karolinska Institutet, 17165 Solna, Sweden
* correspondance: aurelien.barbotin@inrae.fr, rut.carballido-lopez@inrae.fr

Live bacteria

Influence of label concentration on FCS outcome

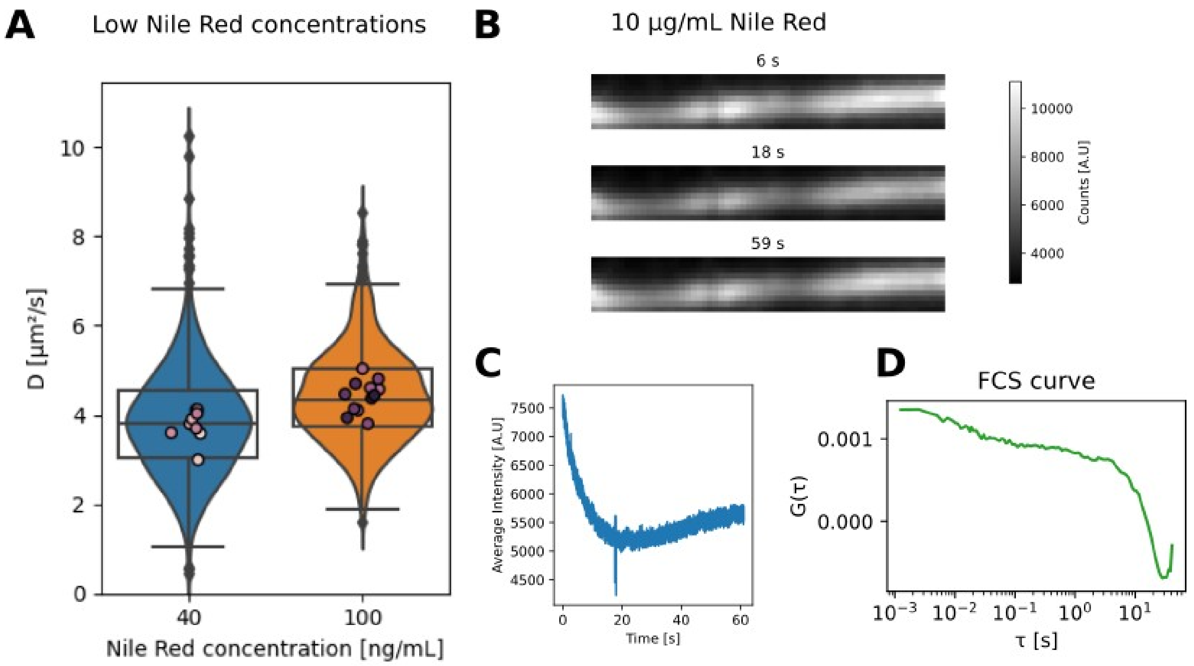

Figure S1 Influence of Nile Red concentration on diffusion coefficient measurement with TIR-FCS in B. subtilis at 37°C. (A) Diffusion coefficient of Nile Red in the membrane of B. subtilis labeled with two different Nile Red concentrations. (B) Average of 1000 frames of a TIR-FCS acquisition in B. subtilis labeled with 10 µg/mL Nile Red concentration, after 6 (top), 18 (middle) and 59 (bottom) seconds of acquisition. (C) Time-dependent intensity averaged across all pixels of the TIR-FCS acquisition shown in (B). (D) representative FCS curve obtained from the acquisition shown in (B)

We tested whether the concentration of membrane marker influenced the outcome of TIR-FCS experiments. For this, we performed TIR-FCS measurements of the diffusion coefficient of Nile Red in the membrane of exponentially-growing *B. subtilis* cells at 37°C, labelled with either a final concentration of 40 or 100 ng/mL of Nile Red (Fig. S1A). We found similar diffusion coefficients, suggesting that at low concentrations, labeling concentration does not affect the outcome of FCS measurement, as expected (7). A small (~10%) difference between the two concentrations was most likely due to variations in medium properties, marker aliquot or simply statistical variations. At higher concentrations, however, we noticed that during FCS acquisitions, fluorescence intensity

first decreased as expected because of photobleaching but then quickly increased again (Fig. S1B-C). This in turns created artefacts in FCS curves that prevented reliable diffusion coefficient estimation (Fig. S1D). This unexpected increase in fluorescence intensity was likely caused by a membrane remodelling due to phototoxicity, which increases with label concentration.

Diffusion coefficient of Nile Red in the membrane of *B. subtilis* as a function of time spent on agarose pad.

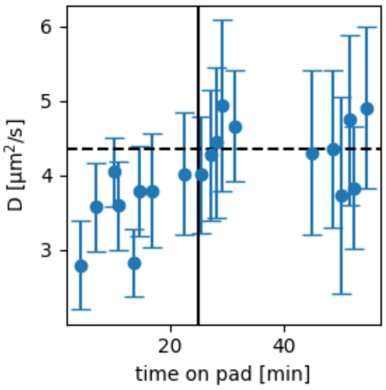

Figure S2: Diffusion coefficient as a function of time spent on an agarose pad at 37°C in growing B. subtilis. Steady-state fluidity is reached after approximately 25 mins (vertical black line), after which imaging can start.

B. subtilis morphology in different conditions

Having found that the width and length of bacterial cells bias diffusion coefficient measurements, we verified whether the morphology of *B. subtilis* cells changed between the different experimental conditions investigated here. For this, we acquired epifluorescence images for each of these conditions (Fig. S3A-B) and measured cell length and width manually using ImageJ. Cell length was determined by drawing a line between the two poles of each cell and measuring the length of this line. We found that the average cell length was identical at 37°C and immediately after cold shock, decreased after 5hours at 20°C (Fig. S3C), but not to a point where cell length biased FCS measurements.

Cell width was measured by plotting the intensity profile alongside a line orthogonal to the cell long axis and measuring the peak-to-peak distance. This method led to an underestimation of the real cell width due to off-axis fluorescence emitted by the top and bottom part of the membrane, hence the relative difference with the well-known diameter of *B. subtilis* of 0.9-1µm (3-4). It revealed however that as expected cell width did not change significantly between experimental conditions and thus that we could apply the same correction factor accounting for membrane curvature to measurements (Fig. S3D). Cell width remained constant during cold shock (Fig. S3F) and cell length remained well above the 2.5 µm threshold leading to bias in diffusion coefficient (Fig. S10A). Panels C and D of Fig. S3 were generated using supplementary ref 1.

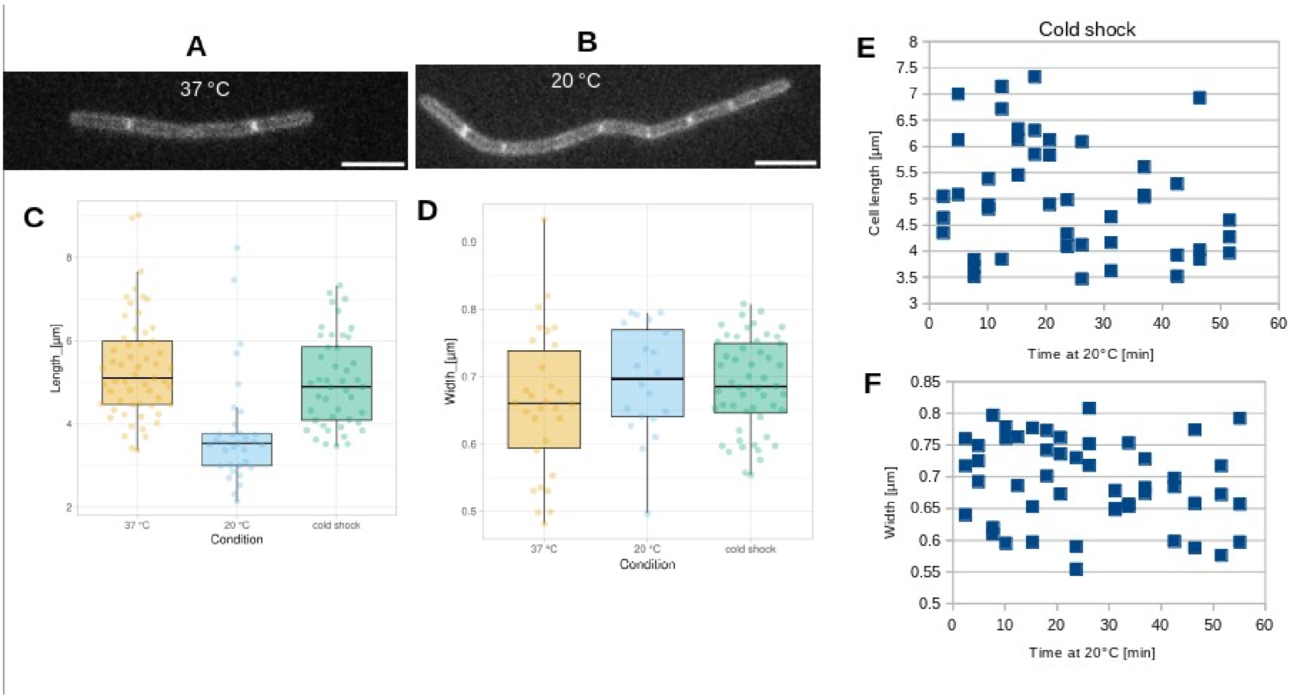

Figure S3: Morphology of B. subtilis at different temperatures. (A-B) epifluorescence images of B. subtilis in exponential phase, labeled with Nile Red, at 37°C (A) and 20 °C (B). Scalebars: 5 µm. (C-D) Length (C) and width (D) of B. subtilis measured from epifluorescence images, in exponential phase at 37°C, 20°C, or during cold shock. (E-F) Scatterplots of cell length (E) and width (F) with time at 20°C immediately after cold shock.

Impact of FCS measurement on doubling time in *B. subtilis*

Using bright-field timelapses, we verified both cell fitness and the impact of phototoxicity on cell growth. For this, we measured the growth rate of cells used in Fig. 3C, at 37°C. We acquired for each chain of cells 3 bright-field images (Fig. S4A), one at least 3 mins before the beginning of FCS acquisition, one immediately after the FCS acquisition and one at least 3 mins after FCS acquisition. We measured the length of the cell chain in each bright-field image and calculated doubling times between pairs of frames following the equation (under the assumption of constant cell width as is the case in *B. subtilis*):

$$T_{double} = \Delta t \ln(2) / \ln(l_2 / l_1) \qquad 1$$

Where T_{double} is the doubling time, Δt is the time between frames 1 and 2, l_1

and l_2 are the lengths of the cell chain in frames 1 and 2. Cells which doubling was more than twice higher than the nominal doubling time (~20 mins) were considered not exponentially-growing and therefore excluded from the analysis. Comparing pairwise doubling times before and after FCS (Fig. S4B), we found that cells kept growing after FCS, yet at a slightly slower rate, suggesting low photoxicity effects.

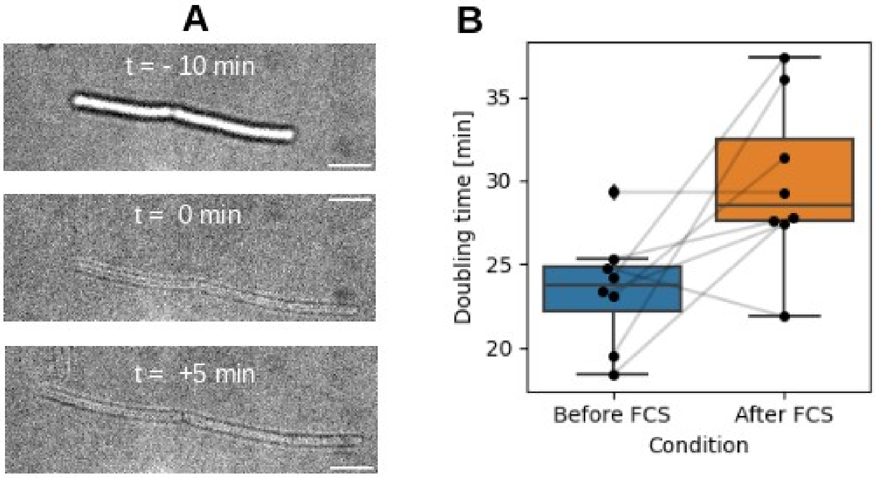

Figure S4: Impact of FCS measurements on the growth rate of Nile Red-labeled B. subtilis cells. (A): bright-field images of growing cells acquired before (top), immediately after (middle) and after (bottom) FCS acquisition. Scale bars: 5 µm. (B) Doubling times calculated from cell elongation, before and after FCS acquisition. Black dots: single doubling times measurements, gray lines link doubling times of the same cell chain.

S. aureus morphology in different conditions

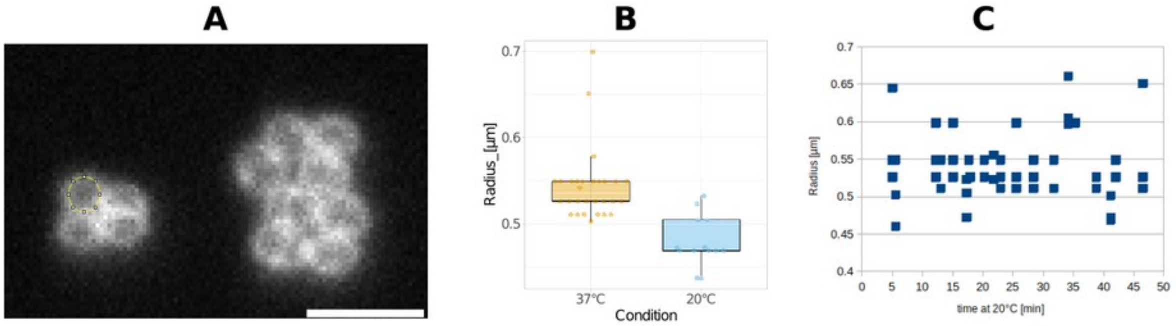

Figure S5 Measuring cell radius of S. aureus at different temperatures. (A) Cell area is measured by manually fitting an ellipsoid (yellow) to epifluorescence images of S. aureus cells labeled with Nile Red. Scale bar: 3 µm. Cells diameter is extracted from area either in a steady-state (A) or during a cold shock (B).

Membrane fluidity in bacteria

We corrected the bias induced by the curvature of S. aureus cells using simulations, assuming that S. aureus cells were spheres of diameter 500 nm. We verified this experimentally in all our experimental conditions using images of S. aureus cells labeled with Nile Red. The area A of circular cells was extracted using ImageJ by manually fitting an ellipsoid (Fig. S5A) to the membrane of cells, then their radius R was estimated using the formula $R=\sqrt{(A/\pi)}$. Our results confirmed that the radius of S. aureus cells was indeed ~500 nm in all our imaging conditions.

Individual cold shock replicates in staphylococcus aureus

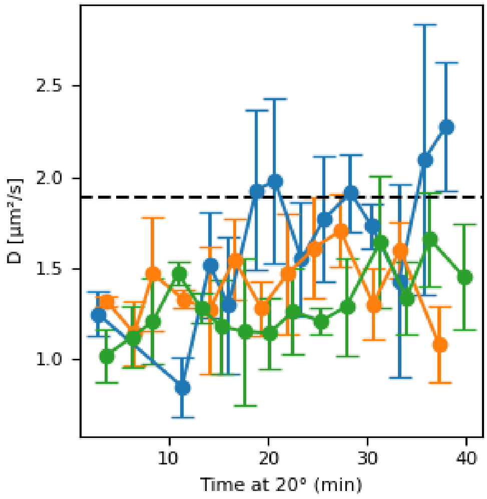

Figure S6: Diffusion coefficient of Nile Red in staphylococcus aureus after transfer at 20°C. Median +/- std of individual acquisitions in the 3 different replicates. Dotted black line: steady-state diffusion coefficient at 20°C.

Detailed implementation of FCS data processing

Intensity threshold

In order to avoid biasing diffusion measurements, we needed to exclude TIR-FCS measurements that were too far from the point of contact between the bacterial cell and the coverslip. An efficient way of doing this consisted in removing pixels with an average intensity below a given threshold, as the excitation of the TIRF field decreases with increased distance to the cell centre. To find an appropriate value for this intensity threshold, we plotted a 2D histogram of intensity (normalised with 98th percentile) and diffusion coefficient in 6 acquisitions of exponentially-growing *B. subtilis* labeled with Nile Red at 20°C. We set the threshold to 0.8 so that there was no correlation between diffusion coefficient and intensity (Fig. S7). We kept the same threshold for *S. aureus* cells. In S. aureus, we applied this intensity threshold not to the whole image but to individual cells, in order to avoid biasing results when one or more cells was brighter than the others in a field of view. The outlines of individual cells were found automatically using a watershed algorithm.

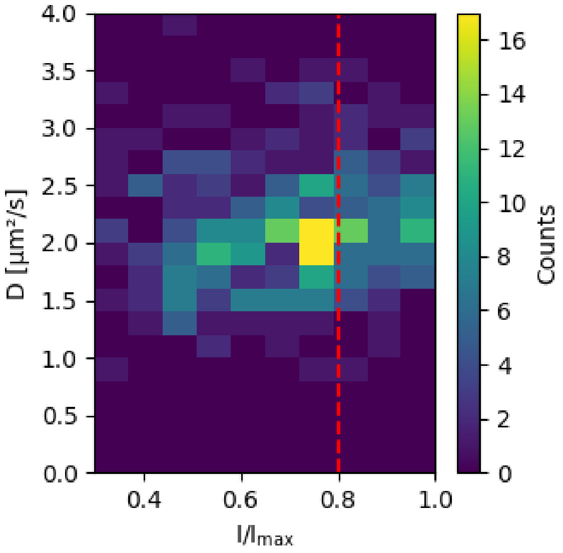

Figure S7: Determination of intensity threshold for unbiased diffusion measurement. Correlation between relative pixel intensity (x axis) and measured diffusion coefficient (y axis) visualised as a 2-dimensional histogram in 6 acquisitions of exponentially-growing B. subtilis at 20°C. Vertical dotted red line: selected threshold

Bleaching correction and FCS fitting

Bleaching correction was performed using a double exponential fit of the decaying intensity. Intensity timetraces were downsampled 500 times to speed up computations. The resulting traces were fitted with the function:

$$\hat{I}(t)=f_0\left[(1-b)\exp(-t/\tau_1)+b\exp(-t/\tau_2)\right]+c \quad 2$$

The original intensity timetrace was then corrected as described in ref (2):

$$I_c(t)=\frac{I(t)}{\sqrt{\hat{I}(t)/\hat{I}(0)}}+\hat{I}(0)\left(1-\sqrt{\hat{I}(t)/\hat{I}(0)}\right) \quad 3$$

The error function in Eq. 1 is defined as :

$$erf(x)=\frac{2}{\sqrt{\pi}}\int_0^x \exp(-t^2)dt \quad 4$$

Simulations

On a sphere: First a set of points representing individual fluorescent emitters were distributed randomly on a sphere, as described in (5). The position of each point was described in spherical coordinates. Its azimuthal (θ) and polar (φ) angles were randomly generated using the following equation:

$$\theta = 2\pi u$$
$$\phi = \cos^{-1}(2v - 1) \quad 5$$

where u and v are drawn from uniform random variables with bounds [0,1[. Trajectories were then converted to cartesian coordinates. At a given time t, the vector position $\vec{r}(t) = [x(t), y(t), z(t)]$ of a point was then updated as follows, as discussed in reference (6):

$$\vec{r}(t+1) = \frac{R}{\|\vec{r}(t) + \vec{r}(t) \wedge \vec{b}(t)\|} (\vec{r}(t) + \vec{r}(t) \wedge \vec{b}(t)) \quad 6$$

where R is the radius of the sphere and $\vec{b}(t) = [u_x(t), u_y(t), u_z(t)]$ is a three-dimensional random vector drawn from a normal distribution, with each component having a standard deviation of $\sqrt{2Dt}/R$, with D the diffusion coefficient. The normalisation factor $\frac{R}{\|\vec{r}(t) + \vec{r}(t) \wedge \vec{b}(t)\|}$ is necessary to keep the vector $\vec{r}(t+1)$ on the surface, as the vector $\vec{r}(t) \wedge \vec{b}(t)$ is tangential to the curved surface and therefore $\vec{r}(t) + \vec{r}(t) \wedge \vec{b}(t)$ is not on the surface (Fig. S8A). Under the conditions that the angle between $\vec{r}(t)$ and $\vec{r}(t+1)$ is small, $\|\vec{r}(t+1) - \vec{r}(t)\| = \vec{r}(t) \wedge \vec{b}(t)$ and the simulation of brownian motion is accurate.

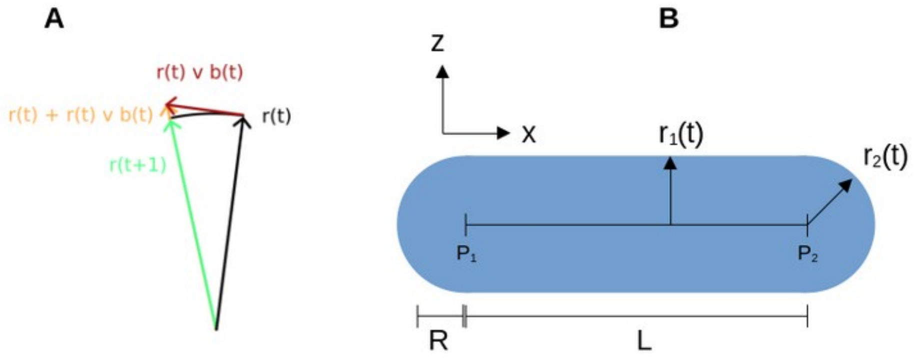

Figure S8: Sketch of the simulation of a Wiener process on curved surfaces. (A): iteration of the Wiener process. B: Sketch of rod-shape simulation.

On a rod: the simulation process is very similar. The rod is represented as a cylinder of length L and radius R with two half-spherical parts or radius R at its end (Fig. S8B), oriented along the x-axis. The initial distribution of points is done in two steps: a fraction of the total number of the points is distributed on a sphere, while the rest of the points are distributed on a cylinder. The relative fraction of points on the sphere and the cylinder is determined from the relative areas of the spherical and cylindrical parts of the rod. Points drawn on the sphere with a negative x coordinate are moved along the x axis by a distance -L/2, the others by a distance +L/2. The vector position $\vec{r}(t)$ of each point is then iteratively updated following Eq. 6 as in the previous section, except that in this case the vector $\vec{r}(t)$ describes the distance to the medial axis (segment [P_1P_2] in Fig.

S8B) of the rod and not to the centre of the sphere. Fig. S8B illustrates the two different configurations ($\vec{r}_1(t)$ and $\vec{r}_2(t)$) for the vector $\vec{r}(t)$.

Parameters used in the simulations of Fig. 2 are listed in the following table:

**Frame rate (kHz)**	**D [µm²/s]**	**# frames**	**Parts. density [parts/µm²]**	**Length [µm] (rod only)**	**Pixel size [µm]**	**σ_{psf} [µm]**	**δz_{TIRF} [µm]**	**Brightness (Hz)**
1	1	50000	1.6	3	0.08	0.19	0.1	20000

Particle density was set to the constant value of 1.6 particle/µm², except in smallest simulations for which it was increased to contain at least 10 particles. TIRF penetration depth δz was defined as:

$$I_{TIRF}(z)=I_0\exp(-z/\delta z) \qquad 7$$

Where I(z) is the depth-dependent TIRF excitation field. To speed up calculations, all particles above 4δz were considered to have a brightness equal to zero and were discarded from the analysis. Analysis was performed with 4x4 binning to an observation area of 320 nm, similar to the one we used in our experiments. An intensity threshold set to 80% of the maximum intensity was also used to analyse simulations as we used in real experiments. Each simulation was performed 9 times. The lateral position of the simulated bacterium was different for each of the 9 simulations to avoid a potential bias.

Influence of size of closedness of the system:

In order to understand if the closedness of the simulated systems described above and in Fig. 2 could lead to a bias in diffusion coefficient estimation with imFCS, we simulated a simple system of 2-dimensional Brownian motion in a homogeneous illumination field. Molecules leaving the system on one edge were reintroduced at the corresponding position on the opposite edge (Fig. S9A). When the box became very small, we could observe that FCS curves shifted towards shorter lag times and became distorted (Fig. S9B). Fitting curves for different box sizes to extract diffusion coefficients confirmed that smaller box sizes, of areas in the order of magnitude of bacterial membrane areas, indeed induced a bias in diffusion coefficient estimation (Fig. S9C). It is therefore very likely that part of the measurement biases in Fig. 2 were caused by an effect of the small size of the systems observed.

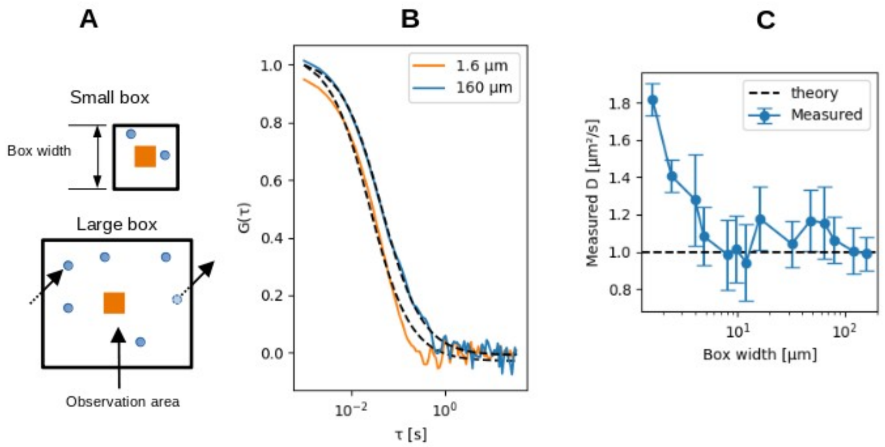

Figure S9: TIR-FCS simulations in a closed box of varying size. (A) sketch of the simulated system with box (black) containing moving particles (blue) and a smaller observation area (centre, orange square) where TIR-FCS is simulated. Top: small box, bottom: large box. A molecule leaving on one side and reentering on the other side is shown with dashed arrows. (B) representative FCS curves obtained when a small (1.6 µm width, orange) and large (160 µm width, blue) simulation box are used. (C) Measured diffusion coefficients in simulated TIR-FCS experiment as a function of box size.

Influence of rod length and PSF size:

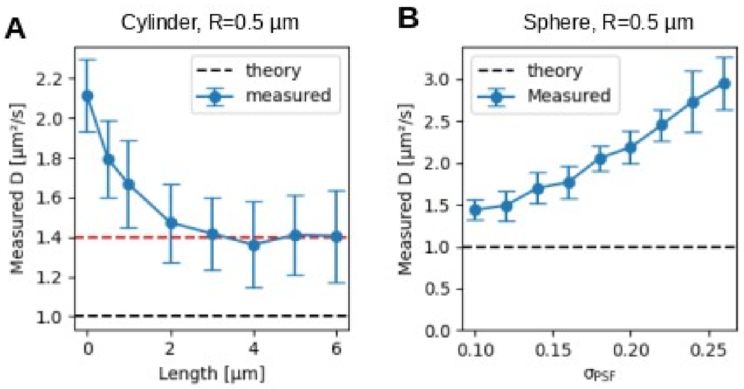

Figure S10: Simulation of the influence of physical parameters on measured diffusion coefficient. (A) Measured diffusion coefficient with length of rod-shape, for a radius of 0.5 µm. Red dashed line: bias for lengths>2.5 µm. (B) Influence of PSF size (parameter σ in Eq. 1-2) on measured diffusion coefficient, on a sphere of radius 0.5 µm.

Supporting References :

1. Lord, S. J.; Velle, K. B.; Mullins, R. D.; Fritz-Laylin, L. K. SuperPlots: Communicating Reproducibility and Variability in Cell Biology. *Journal of Cell Biology* **2020**, *219* (6), e202001064. https://doi.org/10.1083/jcb.202001064.

2. Ries, J., S. Chiantia, and P. Schwille. 2009. Accurate Determination of Membrane Dynamics with Line-Scan FCS. *Biophysical Journal*. 96:1999–2008.

3. Errington J, Aart LTV. 2020. Microbe Profile: *Bacillus subtilis*: model organism for cellular development, and industrial workhorse. Microbiology (Reading). May;166(5):425-427.

4. Juillot, D., C. Cornilleau, N. Deboosere, C. Billaudeau, P. Evouna-Mengue, V. Lejard, P. Brodin, R. Carballido-López, and A. Chastanet. 2021. A High-Content Microscopy Screening Identifies New Genes Involved in Cell Width Control in Bacillus subtilis. 6.

5. https://www.bogotobogo.com/Algorithms/uniform_distribution_sphere.php

6. https://math.stackexchange.com/questions/3725288/infinitesimal-generator-of-the-brownian-motion-on-a-sphere

7. Schneider, F., P. Hernandez-Varas, B. Christoffer Lagerholm, D. Shrestha, E. Sezgin, M. Julia Roberti, G. Ossato, F. Hecht, C. Eggeling, and I. Urbančič. 2020. High photon count rates improve the quality of super-resolution fluorescence fluctuation spectroscopy. *J. Phys. D: Appl. Phys.* 53:164003.
